# Bromide-mediated pathway modulation enables energy-efficient electrosynthesis of ultralight hierarchical copper powder

**DOI:** 10.1039/d6ra04989c

**Published:** 2026-08-21

**Authors:** Keshu Song, Youpo Mise, Shaohua Wang, Yakun Yin, Juan An, Xuejiao Zhou, Xiaoli Yuan, Wentang Xia, Wenqiang Yang

**Affiliations:** a School of Metallurgy and Power Engineering, Chongqing University of Science and Technology Chongqing 401331 P.R. China wenqiangyang@cqust.edu.cn +86-023-65023711 +86-023-65023711; b Chongqing Municipal Key Laboratory of Institutions of Higher Education for Value-added Treatment and Green Extraction from Complicated Resources Chongqing 401331 P.R. China

## Abstract

This study presents a bromide-modulated electrolytic route for producing ultralight copper powder (UCP) through the coupled regulation of copper deposition behavior and hierarchical microstructure evolution. Electrochemical measurements, potential-dependent phase identification, and time-resolved *ex situ* characterization support the transient participation of CuBr-rich species during Br^−^-modified copper deposition. The results are consistent with a proposed additional CuBr-mediated route involving Cu^2+^ → CuBr-rich species → Cu^0^ that operates alongside direct Cu^2+^ reduction, thereby altering the nucleation and growth behavior of copper deposits. Based on the observed temporal evolution, a four-stage growth model is proposed: (i) formation and assembly of CuBr-rich particles, (ii) progressive disappearance and structural collapse of the early deposits under continued cathodic polarization, (iii) emergence and fractal reconstruction of Cu-rich particles, and (iv) diffusion-dominated growth of fern-like dendrites. The resulting UCP exhibits an ultralow apparent density of 0.18 g cm^−3^ and a specific surface area of 4.9 m^2^ g^−1^, which is 6.1 times that of a commercial copper powder reference, owing to the multiscale porosity of its fractal dendritic architecture. This hierarchical structure also imparts a static water contact angle of ∼151°, consistent with air retention at the interface. In antibacterial tests, 0.50 g L^−1^ UCP-30Br with 12 h exposure reduced culturable *Escherichia coli* and *Staphylococcus aureus* to below the detection limit. This efficacy is consistent with enhanced physical contact and interfacial interactions enabled by the hierarchical architecture, coupled with a higher endpoint concentration of soluble copper. Notably, the Br^−^-modified process achieved a current efficiency of 89.7% and a specific DC energy consumption of 1050.9 kWh t^−1^ under the optimized conditions, representing a 21.9% decrease relative to the Br^−^-free control.

## Abstract 2

This study presents a bromide-modulated electrolytic route for producing ultralight copper powder (UCP) through the coupled regulation of copper deposition behavior and hierarchical microstructure evolution.

## Graphical abstract

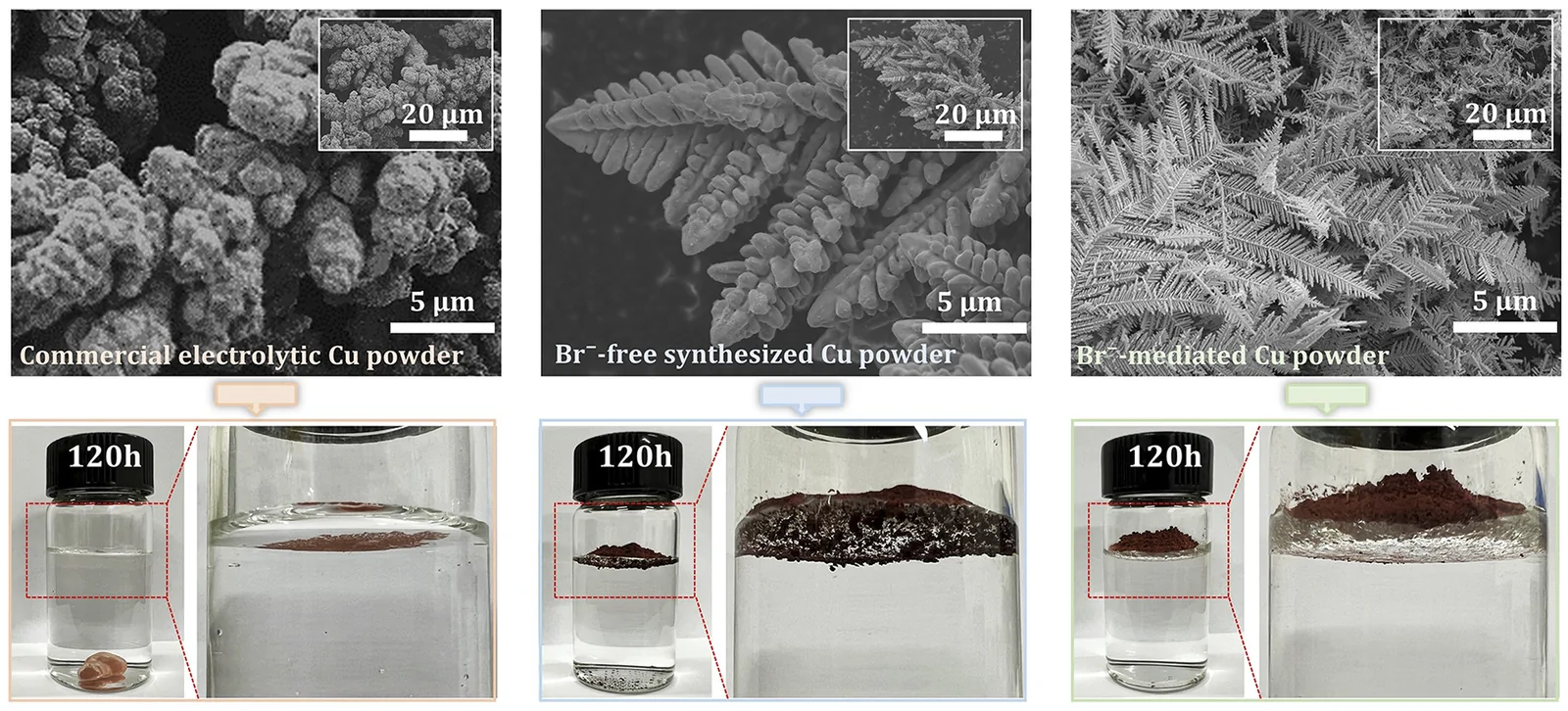

## 1 Introduction

Three-dimensional (3D) micro/nano metallic materials, particularly those featuring fractal, dendritic, or ordered porous architectures, have garnered significant attention due to their exceptional specific surface areas and interconnected pore networks.^1–3^ These structural characteristics confer distinct advantages for enhancing interactions at the electrode-gas/liquid interface and improving electron transport,^4,5^ making them highly suitable for applications in batteries,^6^ sensors,^7^ antimicrobial materials,^8,9^ superhydrophobic surfaces,^10^ supercapacitors,^11^ and catalysis.^12^ Among these materials, ultralight copper powder (UCP) stands out due to its intricately branched dendritic morphology and ultralow apparent density (<1.0 g cm^−3^). The dendritic structure of UCP directly correlates with its specific surface area, where more developed dendrites result in lower apparent density and higher surface area. Therefore, UCP excels not only in the aforementioned applications but also, owing to its superior electrical and thermal conductivity, serves as a preferred material for advanced specialized fields such as electronic components, precision brushes, and electromagnetic shielding.^13–15^

Electrochemical deposition remains the sole method for large-scale UCP production, valued for its product quality, production efficiency, process controllability, and cost-effectiveness.^16,17^ However, industrial UCP production faces challenges in achieving highly dendritic morphologies and ultralow apparent densities while simultaneously maintaining high production efficiency. Conventional electrolysis parameter control strategies struggle to reconcile the competing demands for fine particle size and well-developed dendritic architectures within an economically viable energy consumption range.^18^ To address this limitation, electrolyte additives have been introduced as a critical strategy for fine-tuning deposition kinetics. Additives can modulate interfacial reactions through mechanisms such as cathode adsorption, complexation with metal ions, and control of ion diffusion,^19,20^ thereby optimizing copper powder properties. For instance, anionic polymers can be adsorbed onto copper nuclei, forming a protective layer that inhibits excessive grain growth,^21,22^ while chloride ions promote preferential growth along the (110) plane through complexation with copper ions.^23^ Beyond electrolytic copper powder, the structure-directing role of additives in other copper-based materials has also been extensively validated, including the use of hydrochloric acid for refining copper foam,^24^ ethylenediaminetetraacetic acid for boosting CO_2_ electroreduction efficiency by optimizing intermediate adsorption,^25^ and cetyltrimethylammonium bromide for creating hollow copper-based MOFs.^26^ However, research specifically targeting the additive-mediated synthesis of UCP remains scarce. Consequently, exploring specific additive strategies offers a promising avenue to overcome current manufacturing limitations and achieve precise structural regulation of UCP.

Here, we report a bromide-modulated electrochemical approach that addresses the trade-off between energy efficiency and structural quality in UCP manufacturing. By systematically investigating four electrolyte additives—citric acid, glycine, hydrobromic acid, and acetic acid—we show that bromide ions markedly alter copper deposition behavior, enabling the production of highly dendritic UCP with an ultralow apparent density while maintaining high current efficiency and significantly reduced energy consumption. Electrochemical measurements and multiscale time-resolved characterization support a proposed CuBr-mediated pathway and provide insights into the hierarchical growth of the resulting fractal structures. The obtained UCP further exhibits antibacterial activity and a high apparent static water contact angle. This work thereby provides a viable and efficient strategy that balances structural control with energy efficiency.

## 2 Results and discussion

### 2.1 Additive-dependent modulation of UCP morphology, microstructure, and production efficiency

**Fig. 1:**
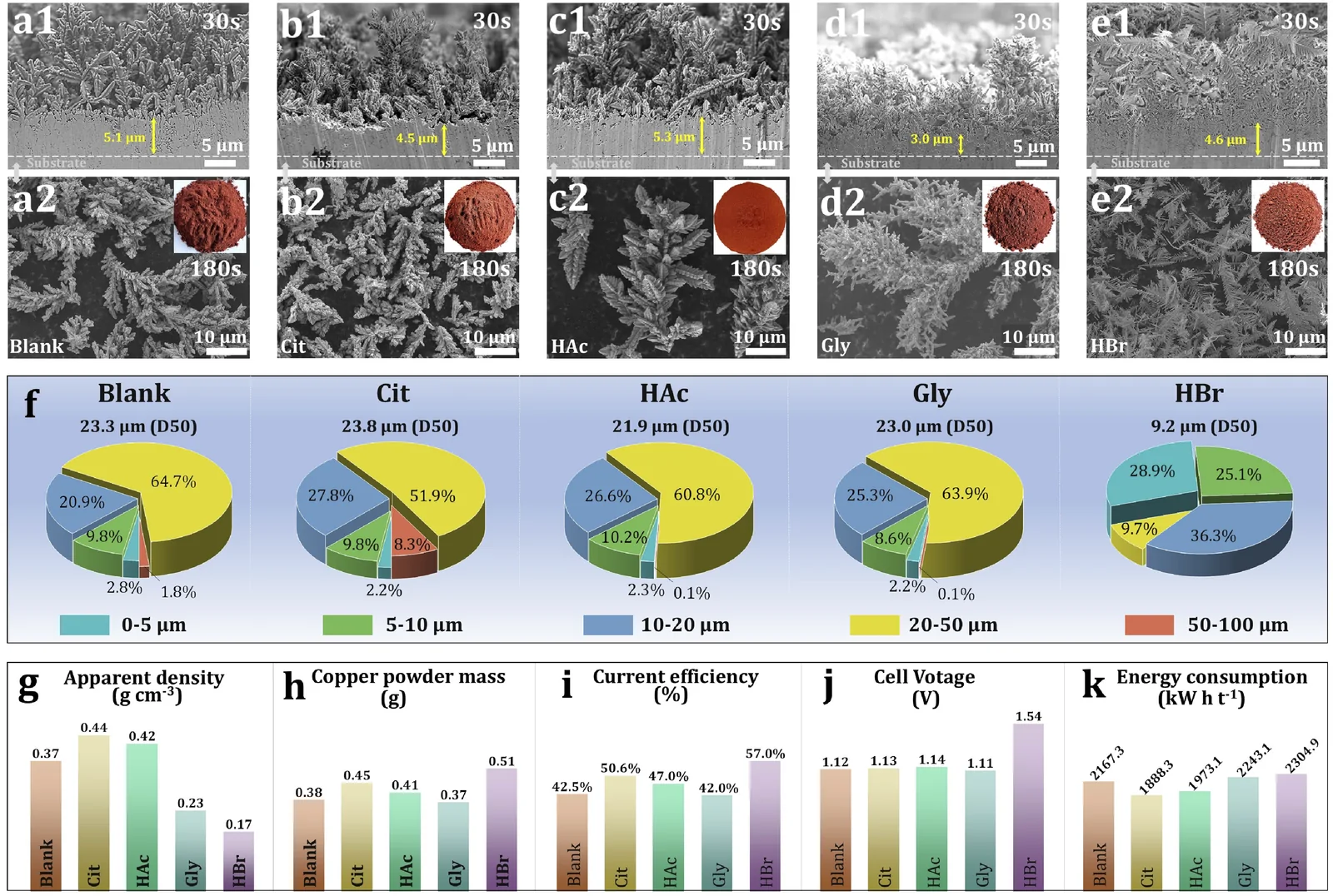
Characteristics of copper powders produced from electrolytes with various additives (as indicated) under condition A (Table S1): (a1–e1) SEM images of cross-sections of copper after 30 s of deposition. (a2–e2) SEM images of scraped copper powders after 180 s of deposition, and their (f) particle size distribution, (g) apparent density, (h) powder mass, (i) current efficiency, (j) cell voltage, and (k) power consumption.

To overcome the limitations of conventional industrial UCP production, particularly in controlling dendritic morphology and product quality, this study implemented an additive-mediated strategy. The additives—citric acid (Cit), glycine (Gly), hydrobromic acid (HBr), and acetic acid (HAc)—were selected for their distinct interfacial modulation mechanisms, where Cit acts as a strong complexing agent,^27,28^ Gly serves as a bidentate ligand,^29,30^ HBr represents a halide-ion mediator, and HAc is included as a weakly coordinating ref. 31 and 32 systematic investigation revealed their distinct impacts on UCP characteristics and production efficiency, as evidenced by comprehensive morphological analysis (Fig. 1a–e and S3). The additive-free control group exhibited typical 3D dendritic clusters with internal honeycomb porosity (Fig. S3a), which resulted from hydrogen co-deposition.^33^ Cross-sectional analysis demonstrated a well-defined tri-layered architecture: (i) a dense basal layer (∼5.1 µm) exhibiting strong substrate adhesion, (ii) an intermediate transition zone of columnar crystal layer, and (iii) an outer dendritic layer demonstrating characteristic sorghum-like morphology (Fig. 1a and S4). This hierarchical configuration facilitates the detachment of the outer loose structures during powder harvesting, yielding coarse dendritic aggregates with disordered, less-fractal branching. However, the robust adhesion of the dense basal layer to the substrate can lead to the formation of cathode residues.

Additive incorporation maintained the tri-layered structure while significantly modifying layer morphology and thickness (Fig. 1b–e). Specifically, Cit reduced the thickness of the basal layer to ∼4.5 µm while exacerbating dendritic disorder and suppressing fractal growth (Fig. 1b and S5). HAc induced a cauliflower-like morphology with improved fractal order but increased basal thickness (∼5.3 µm) (Fig. 1c and S6). In contrast, Gly optimized dendritic architecture, producing fine, uniform coral-like structures with reduced basal thickness (∼3.0 µm) (Fig. 1d and S7). Notably, HBr significantly increased the density of hydrogen evolution pores while reducing pore diameter (yellow circle, Fig. S3e), concurrently promoting the formation of highly ordered, fractal fern-like dendrites. This morphological refinement was accompanied by a decrease in dense layer thickness to ∼4.6 µm (Fig. 1e and S8).

Particle characteristics and energy efficiency exhibited distinct additive-dependent variations (Fig. 1f–k). The additive-free system yielded powders with a broad particle size distribution (0–63 µm), characterized by a D50 of 23.3 µm and an apparent density of 0.37 g cm^−3^. Cit adversely affected these properties, whereas HAc slightly narrowed the size distribution but increased apparent density (0.42 g cm^−3^). In contrast, Gly achieved concurrent refinement, significantly narrowing the distribution and reducing apparent density (0.23 g cm^−3^). Most notably, HBr exhibited optimal performance, producing powders with 90.3% of particles below 20 µm, coupled with the lowest D50 (9.2 µm) and apparent density (0.17 g cm^−3^). While HAc and Gly showed negligible improvements in efficiency or energy consumption, HBr significantly enhanced output and achieved the highest current efficiency, despite a 6.3% energy consumption increase, likely attributed to the formation of white deposits on both electrodes (Fig. S9; Videos S1 and S2). Particularly, Cit demonstrated superior energy efficiency, boosting yield by 18.4%, raising current efficiency by 19.1%, and reducing energy consumption by 12.9%.

The effect of additives on the crystal structure and purity was further examined. XRD patterns (Fig. S10a) confirmed that all samples exhibited face-centered cubic (FCC) copper (JCPDS card No. 04-0836), indicating that the crystalline phase was pure copper. EDS mappings (Fig. S10b–f) detected trace oxygen across all powders, attributable to natural surface oxidation. While UCPs produced with Cit, HAc, and HBr exhibited negligible impurities, the Gly-assisted sample showed a localized nitrogen signal (1.28 at%). To accurately quantify the bulk impurity level in the Gly-assisted sample, elemental combustion analysis was conducted. The result revealed low bulk oxygen and nitrogen contents of 0.22 wt% and 0.020 wt%, respectively. This result confirms that the nitrogen detected by EDS is highly localized, likely present as surface adsorbates or trapped within dendritic occlusions, rather than incorporated into the copper matrix. This interpretation is further supported by cross-sectional EDS analysis (Fig. S11), which showed an absence of nitrogen within the dense layer. Collectively, these analyses verify that the oxygen signal originates from superficial oxidation, and the nitrogen is not a bulk dopant.

**Fig. 2:**
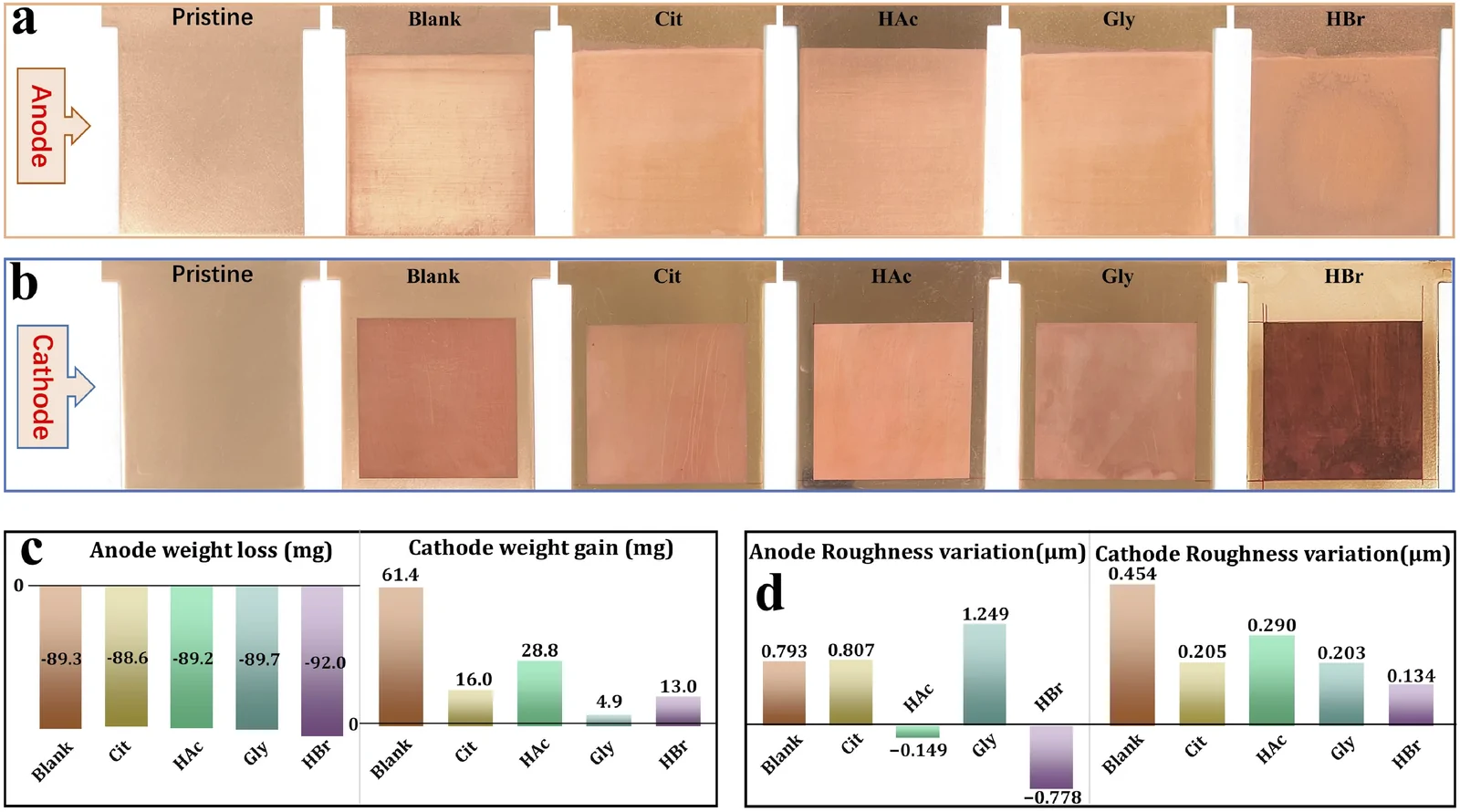
Surface morphology and characteristics of anodes and cathodes after 180 s of electrodeposition under condition A (Table S1) with different electrolyte additives (as indicated) and subsequent brushing treatment. (a) Macroscopic images of anodes, (b) macroscopic images of cathodes, (c) weight loss of anodes and weight gain of cathodes, (d) surface roughness changes of anodes and cathodes.

Electrode surface properties critically govern metal nucleation, growth, and final morphology.^34,35^ Surface characterization of the scraped electrodes deposited in additive-containing electrolytes (Cit, Gly, HAc, or HBr) revealed significant additive-dependent transformations. The additives minimally affected anode macroscopic appearance but significantly modified cathode surface (Fig. 2a and b). For anodes, the additives had negligible effects on mass loss (Fig. 2c), except for HBr, which accelerated anode dissolution (2.7 mg increase). This phenomenon was associated with the formation of white deposits on the anode surface (Fig. S9c).

Conversely, cathodic residue accumulation varied significantly, peaking under additive-free and HAc-modified conditions, while Gly treatment minimized residue to 4.9 mg (Fig. 2c). This variation confirms that the dense basal layer directly contributes to cathode residue formation owing to its strong adhesion to the substrate, which hinders complete deposit detachment (Fig. S4–S8). Furthermore, the layer induces uneven surface development, leading to irregular surface features and morphology deterioration (Fig. 2d and S12). Surface roughness analysis revealed a non-linear correlation between cathodic roughness and residue accumulation, with roughness peaking under additive-free and HAc-added conditions. Gly and Cit treatments reduced cathode roughness by ∼55%, while HBr achieved a ∼70% reduction, minimizing dendritic growth sites and mitigating uneven active site distribution. This optimization suppressed disordered crystal growth and irregular particle formation, thereby enhancing UCP quality and energy efficiency. For anodes, roughness increased by 0.793 µm without additives, with Cit and Gly further exacerbating this trend. In contrast, HBr and HAc reduced roughness by 0.149 µm and 0.778 µm, respectively. Maintaining a smooth anode surface facilitates uniform reactions, extends service life, and minimizes fragment contamination in the copper powder at the electrolytic tank bottom.

### 2.2 Electrochemical behavior of copper deposition in additive-containing electrolytes

Given the significant influence of additives on UCP morphology, structure, and performance, this study investigated the underlying mechanisms. Experiments were conducted using a base electrolyte of 0.08 M CuSO_4_ and 1.53 M H_2_SO_4_, supplemented with 0.15 M Cit, Gly, HBr, or HAc to establish a comparative research framework.

**Fig. 3:**
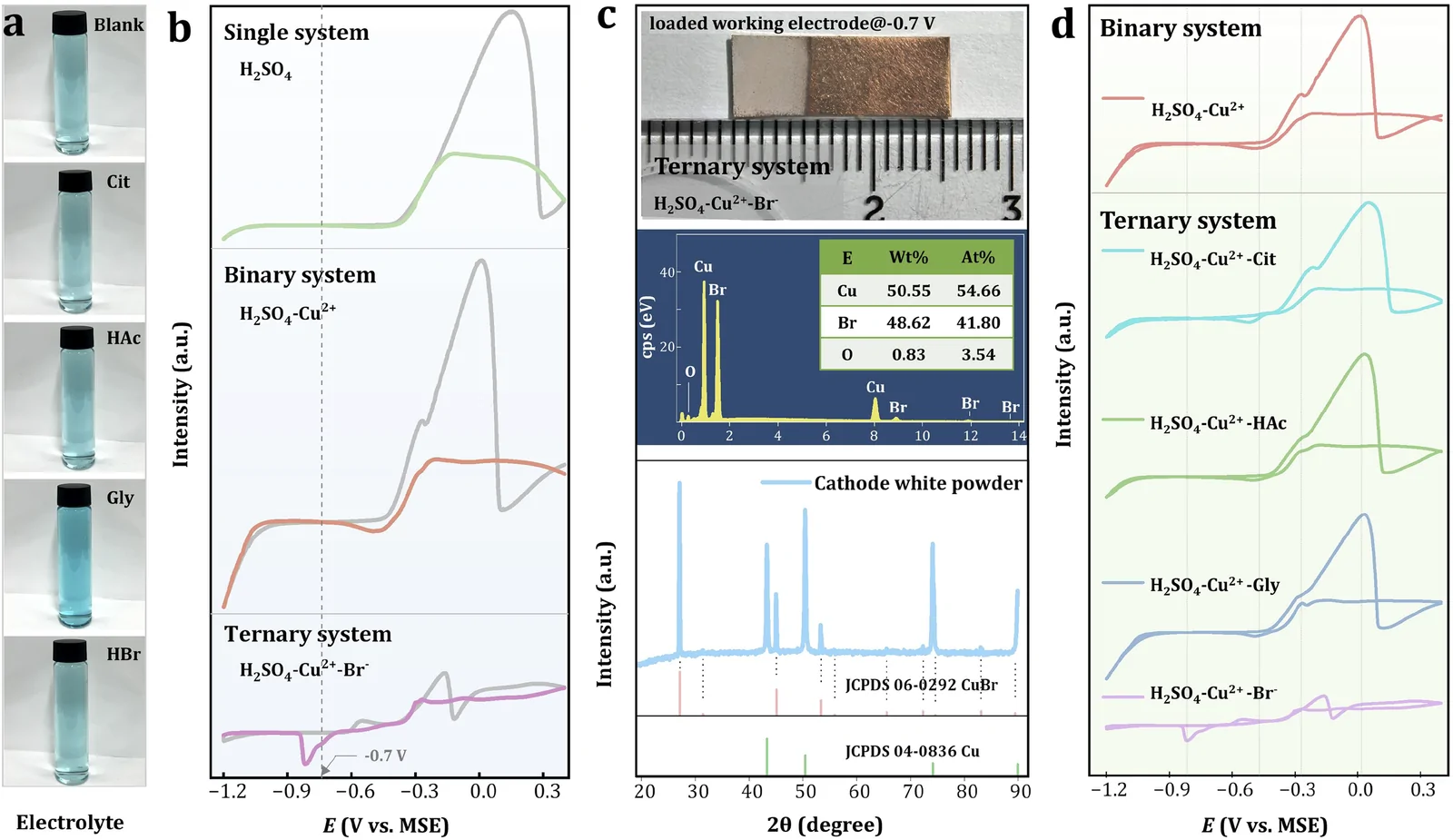
(a) Appearance of electrolyte systems containing 1.53 M H_2_SO_4_, 0.08 M CuSO_4_, and 0.15 M specified additive (as indicated). (b) CV comparison in single (1.53 M H_2_SO_4_), binary (1.53 M H_2_SO_4_–0.08 M CuSO_4_), and ternary (1.53 M H_2_SO_4_–0.08 M CuSO_4_–0.15 M HBr) systems; colored and grey curves represent forward and reverse scans, respectively. (c) Macrographs, EDS, and XRD of the working Cu electrode prepared from the solution containing 1.53 M H_2_SO_4_, 0.08 M CuSO_4_, and 0.15 M HBr at −0.7 V for 2 s per deposition, totaling 15 depositions. (d) CVs of Cu electrodes in the binary and ternary systems (as indicated) at 296 K, scanned at 10 mV s^−1^ within −1.2 to 0.4 V.

The effects of these additives on the coordination state and concentration of Cu^2+^ ions were analyzed using UV-vis spectroscopy, complemented by electrochemical techniques—CV, CA, EIS, and LSV. These methods collectively elucidated the regulatory mechanisms of additives on electrochemical behavior, redox properties, interfacial reaction kinetics, and growth patterns. In the absence of additives, CuSO_4_ dissolution in the electrolyte resulted in hydrated Cu^2+^ ions, imparting a light blue color to the solution (Fig. 3a). UV-vis spectra showed a characteristic absorption peak at 810 nm (Fig. S13), corresponding to the d–d transition of hydrated Cu^2+^ ions.^36^ The addition of HAc and HBr had negligible effects on the electrolyte color or absorption peak position, indicating minimal alteration to the Cu^2+^ coordination environment or concentration. In contrast, Gly deepened the electrolyte color and attenuated the absorption peak, likely due to competitive coordination between its amino and carboxyl groups and Cu^2+^, altering the coordination state or reducing Cu^2+^ effective concentration.^37^ Conversely, Cit significantly intensified the absorption peak, which was attributed to the formation of stable coordination complexes between Cit, a strong ligand, and Cu^2+^.^27,28^

CV analysis revealed that additives significantly influenced the electrochemical redox behavior of copper, with HBr showing particularly pronounced effects. In pure H_2_SO_4_ electrolyte (Fig. 3b), the reduction current onset at −1.01 V marked hydrogen evolution initiation, while the peak at 0.15 V corresponded to copper substrate oxidation. Following CuSO_4_ addition, the Cu^2+^ reduction onset shifted to −0.40 V with a peak current at approximately −0.49 V, representing Cu^2+^ reduction to Cu^0^, accompanied by a Cu^0^ oxidation peak at −0.27 V. Notably, HBr introduction caused a substantial −0.33 V negative shift in the reduction peak with enhanced peak intensity. A new reduction shoulder emerged at −0.65 to −0.76 V, concurrent with white deposit formation on the working electrode. Chronoamperometric deposition at −0.70 V (2 s intervals, 15 cycles) identified these deposits as crystalline CuBr (*K*_sp_ = 6.27 × 10^−9^) *via* EDS and XRD analysis (Fig. 3c), supporting the assignment of the Br^−^-dependent shoulder to the cathodic formation of a CuBr-rich species. Br^−^ addition appears to exert dual modulation on Cu^2+^ reduction: (i) it suppresses or delays direct Cu^2+^-to-Cu^0^ reduction, as reflected by the negative shift in the reduction potential, while promoting the formation of CuBr-rich deposits; and (ii) the formation of these CuBr-rich species may introduce an additional CuBr-mediated route to metallic Cu at more negative potentials (<−0.77 V). Through this mechanism, Br^−^ modifies the interfacial deposition process through the formation of CuBr-rich species, thereby influencing the subsequent reduction, nucleation, and growth behavior of copper. Taken together, these observations support the transient participation of CuBr-rich species in the Br^−^-modified deposition process. The proposed CuBr-mediated route may operate alongside direct Cu^2+^ reduction and influence copper nucleation and growth, thereby contributing to the formation of hierarchical fern-like UCP structures. Specifically, Cu^2+^ reduction is proposed to involve an additional two-step CuBr-mediated route: first, the formation of CuBr-rich species, followed by their net electrochemical consumption under continued cathodic polarization, accompanied by the formation of Cu^0^. This proposed route is consistent with the significant negative shift and intensification of the reduction peak, as well as the appearance of the new reduction shoulder. In parallel, the oxidation peak of Cu^0^ shifted negatively to −0.70 V, consistent with Br^−^-assisted conversion of Cu^0^ to CuBr. The CuBr precipitate covered the electrode surface during anodic polarization, impeding copper-electrolyte contact and attenuating the oxidation peak of the copper substrate (Fig. 3b). Notably, no oxidation current response corresponding to the oxidation of CuBr to Cu^2+^ was detected during the positive potential scan, indicating that CuBr oxidation does not occur within the investigated potential window. This interpretation was further supported by scanning a Pt electrode coated with CuBr powder from −0.7 to 0.4 V, which showed no oxidation current response (Fig. S14). The pronounced asymmetry in reduction and oxidation processes is consistent with the formation of CuBr-rich surface species and the introduction of additional Br^−^-dependent electrochemical processes, although changes in interfacial kinetics and electrode surface structure may also contribute to the observed behavior. In contrast to the significant effects of HBr, HAc and Gly exhibited milder influences on the electrochemical behavior of copper (Fig. 3d), with no significant changes in copper redox potentials. Cit, however, demonstrated a unique regulatory effect, shifting the reduction peak negatively to −0.51 V and the oxidation peak positively to −0.21 V. This indicates a Cit–Cu^2+^ interaction that modulates copper redox processes, likely *via* complex formation altering copper's electrochemical behavior.^38,39^

**Fig. 4:**
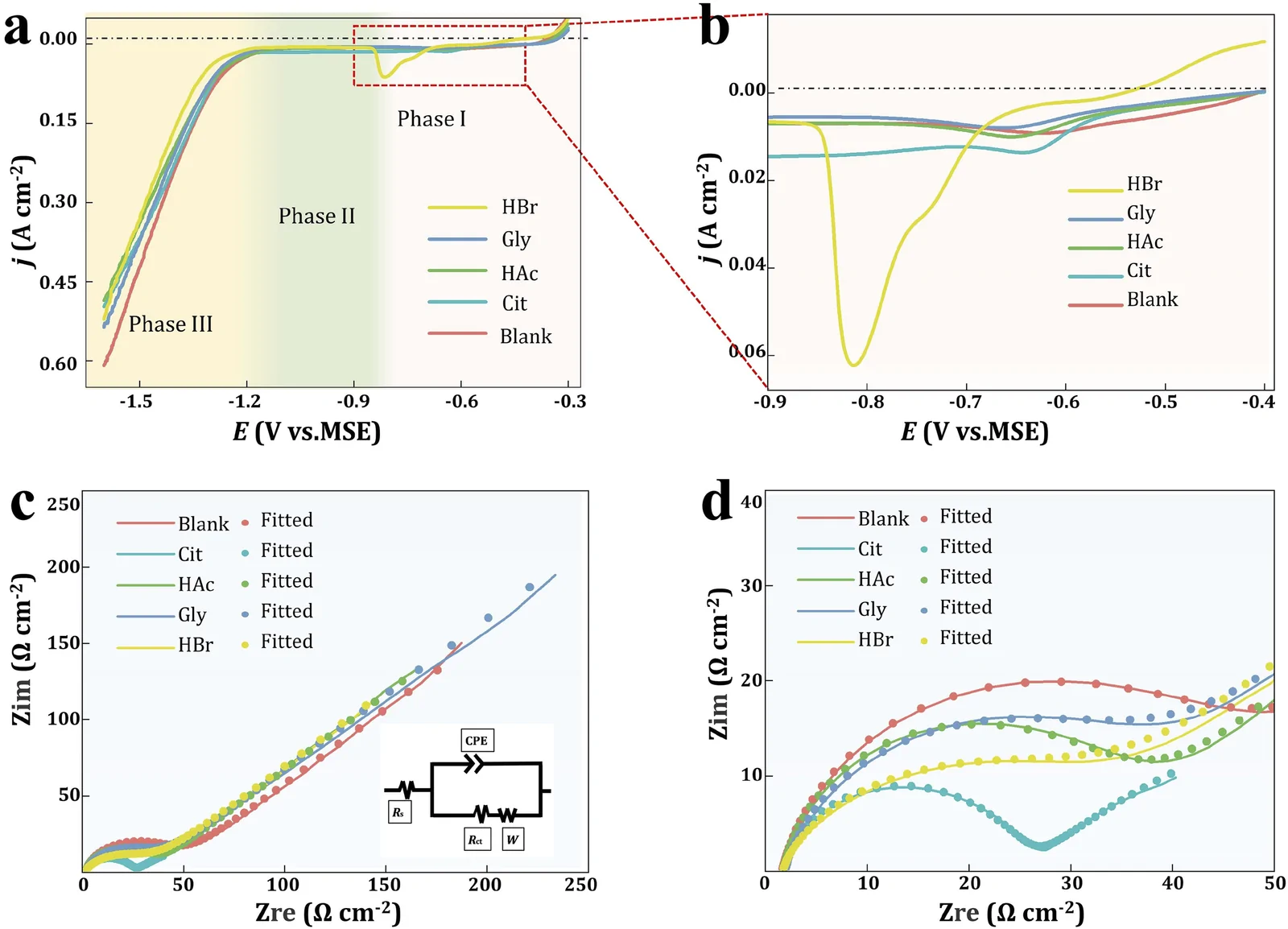
(a) LSV curves and (b) the magnified segment for Cu electrodes in the electrolytes containing 1.53 M H_2_SO_4_, 0.08 M CuSO_4_, and 0.15 M of a specified additive at a scan rate of 5 mV s^−1^. (c and d) Nyquist plots of EIS spectra and the fitted Randles equivalent circuit model for Cu electrodes in the electrolytes used in (a).

The LSV curves (Fig. 4a) provided critical insights into the electrochemical reduction of copper in the presence of various additives. Three distinct phases were evident in the reduction process. Phase I (−0.4 to −0.9 V) represents the exclusive reduction of Cu^2+^, marking the initiation and critical step of copper reduction (Fig. 4b). The potential characteristics of this phase are consistent with the results from the CV tests (Fig. 3b). Phase II (−0.9 to −1.2 V) was characterized by diffusion-controlled kinetics, exhibiting a stable reduction current. Below −1.2 V, the competitive hydrogen evolution reaction (HER) at the cathode signified the initiation of Phase III. As overpotential increased, HER intensified, leading to significant oscillations in the polarization curve between −1.4 and −1.6 V. The additives demonstrated substantial HER suppression capabilities, shifting the onset potentials to more negative values: blank (−1.12 V), HAc (−1.13 V), Cit (−1.14 V), Gly (−1.17 V), and HBr (−1.19 V). Notably, Cit and Gly enhanced cathodic polarization, while HBr showed the most pronounced effect, consistent with the introduction of an additional CuBr-related interfacial process and the formation of a CuBr-rich surface layer on the cathode. This barrier concurrently inhibits both HER and copper reduction, leading to a negative shift in the reduction potential and an elevation in cell voltage, consistent with the observations in Fig. 1j and 3b.

CA analysis was employed to qualitatively explore the kinetic effects of additives on copper electrocrystallization. Given that the deposition occurred on a homogeneous Cu substrate and primarily involved the formation and subsequent reduction of a non-metallic CuBr-rich intermediate phase (Fig. 3c), classical heterogeneous nucleation models were not applied. Therefore, the analysis focused on phenomenological observations. The current–time transient (CTT) curves (Fig. S15), recorded at a step potential of −0.7 V, exhibited three distinct stages of copper deposition: (i) an initial rapid current density drop due to double-layer charging, (ii) a subsequent rise to a peak corresponding to nucleation, and (iii) a subsequent rapid decline to a diffusion-controlled equilibrium state.^40^ In the blank electrolyte and systems containing HAc and Cit, the double-layer charging process was nearly undetectable, likely due to the rapid completion of the process at high potentials. In contrast, the addition of HBr and Gly significantly delayed this process, rendering it clearly observable in the CTTs. The nucleation time in the CA curves varied markedly among the additives: 0.6 s for the blank electrolyte, 0.2 s for Cit, 0.4 s for HAc, and 0.7 s for Gly, indicating that Cit and HAc accelerated copper reduction kinetics, while Gly exerted an inhibitory effect. Notably, HBr exhibited an exceptionally long nucleation time of 5.5 s, as already indicated by the charge-transfer impediment observed in CV and LSV, which was attributed to the formation of a CuBr surface layer (Fig. 3c).

EIS analysis, represented by Nyquist plots (Fig. 4c and d), demonstrated the unique modulation effects of the additives on the impedance characteristics of the electrolyte system. In the high-frequency region, a small semicircle corresponded to the charge transfer process at the electrode–electrolyte interface, where the *x*-intercept represented the solution resistance (*R*_s_) and the semicircle diameter reflected the faradaic charge-transfer resistance (*R*_ct_).^41,42^ The low-frequency region showed an upward-sloping line, corresponding to the diffusion resistance (*W*) and the charge transfer process at the interface. Equivalent circuit fitting of the impedance data (Table S2) highlighted the impact of additives on *R*_ct_: the blank electrolyte exhibited a high *R*_ct_ of 43.77 Ω, while the incorporation of Gly, HAc, and HBr reduced *R*_ct_ to 34.44, 32.91, and 30.97 Ω, respectively. Particularly, Cit achieved the lowest *R*_ct_ of 24.09 Ω, significantly enhancing charge transfer efficiency and optimizing interfacial properties during copper deposition. Collectively, these findings underscore that the rational introduction of additives can markedly improve the charge transfer kinetics at the cathode, thereby enhancing electrolysis efficiency, a conclusion consistent with the results presented in Fig. 1i.

### 2.3 Temporal evolution of electrode interfacial architectures and a proposed bromide-mediated hierarchical growth model

**Fig. 5:**
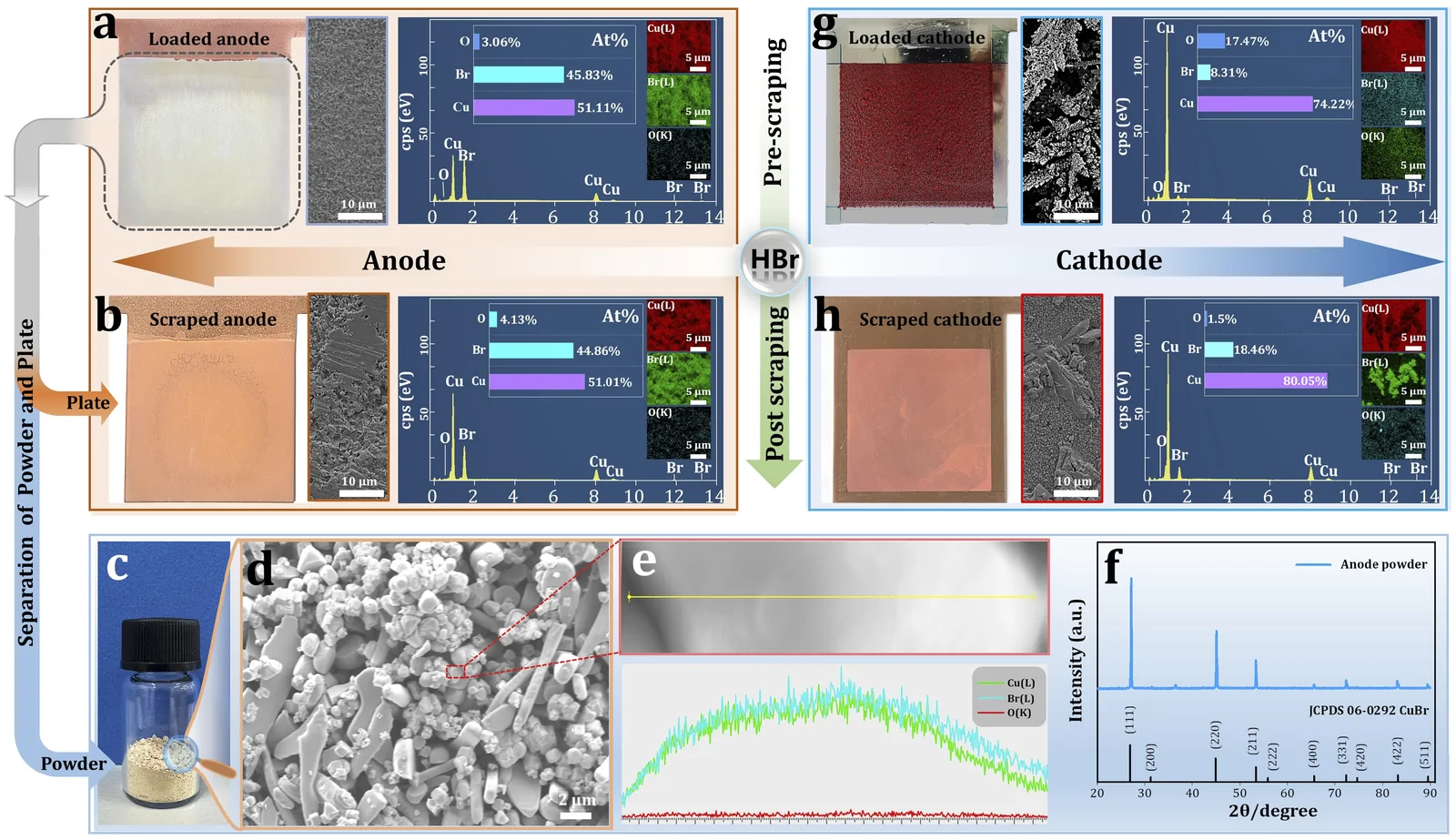
Characterization of electrodes after deposition in the 0.08 M Cu^2+^–1.53 M H_2_SO_4_–0.15 M HBr solution system under condition B (Table S1) for 180 s. Macroscopic morphologies, SEM images and EDS results of (a) the loaded and (b) the scraped anode. (c) Appearance of the scraped powder from anode, and its (d) SEM image, (e) line-scan EDS, and (f) XRD pattern. Macroscopic morphologies, SEM images and EDS results of (g) the loaded and (h) the scraped cathode.

Given the pronounced influence of HBr on UCP deposition characteristics and performance, particularly its distinctive modulation of electrochemical processes and reaction kinetics, this section systematically investigated the temporal evolution of electrode morphologies and mechanistic pathways within the hydrogen bromide electrolyte system. The time-dependent SEM observations were obtained through a series of independent interrupted-electrodeposition experiments under condition B. A freshly prepared Cu anode–cathode pair was used for each electrolysis time of 0.6, 1, 3, 5, 10, and 60 s, with all other operating parameters kept constant. At each predetermined time, the power supply was immediately switched off, and both electrodes were simultaneously removed from the cell and subjected to the same post-deposition treatment before SEM/EDS characterization. At the anode, a unique white deposit rapidly formed during UCP deposition in Br^−^-containing electrolyte, as evidenced by real-time observation (Fig. S16, S17 and Video S1). SEM-EDS characterization revealed that the anode surface was uniformly covered with rough, platelet-like Cu–Br structures, exhibiting a stoichiometric composition of Cu (51.11 at%) and Br (45.83 at%) (Fig. 5a and S18). After removing the white deposit, the anode surface displayed a matte, reddish-copper appearance with microscopically uneven surface (Fig. 5b and S19). Residual blocky structures, chemically similar to the initial deposit, persisted on the surface despite scraping. The collected white material, following washing and drying, formed a pale-yellow powder (Fig. 5c). SEM imaging (Fig. 5d) revealed a heterogeneous mixture of fine particles and large aggregates. EDS line-scan analysis (Fig. 5e) demonstrated a uniform 1 : 1 Cu-to-Br ratio, consistent with prior results (Fig. 5a and b). XRD characterization further confirmed the powder as phase-pure CuBr (Fig. 5f).

On the cathode side, a transient white deposit formed during the initial deposition stage (<3 s) but rapidly disappeared, accompanied by vigorous hydrogen evolution (Fig. S20a and Video S2). EDS analysis revealed that the white deposit consisted of Cu (58.86 at%), Br (37.74 at%), and O (3.40 at%) (Fig. S20b). Together with the phase identification of the deposit formed at −0.70 V (Fig. 3c), this composition is consistent with the formation of a CuBr-rich cathodic deposit. Post-electrolysis, the cathode surface developed a honeycomb-like morphology with hydrogen evolution pores and reddish-brown copper deposits interspersed with white CuBr patches (Fig. S9a and b). Rinsing removed the CuBr, revealing bright red copper deposits (Fig. 5g). Drying further exposed a well-developed fern-like dendritic structure, featuring prominent primary and secondary dendritic arms. Elemental analysis indicated a composition of Cu (74.22 at%), O (17.47 at%), and Br (8.31 at%), with uniform spatial distribution. After removing the surface powder, the cathode exhibited a macroscopically smooth surface with microscopically dispersed irregular particles (Fig. 5h). EDS mapping revealed Br localized exclusively on these particles, while O was faintly distributed, and Cu was enriched on the particle exteriors, suggesting a Cu–Br granular phase, in agreement with CV analysis results (Fig. 3b and c).

**Fig. 6:**
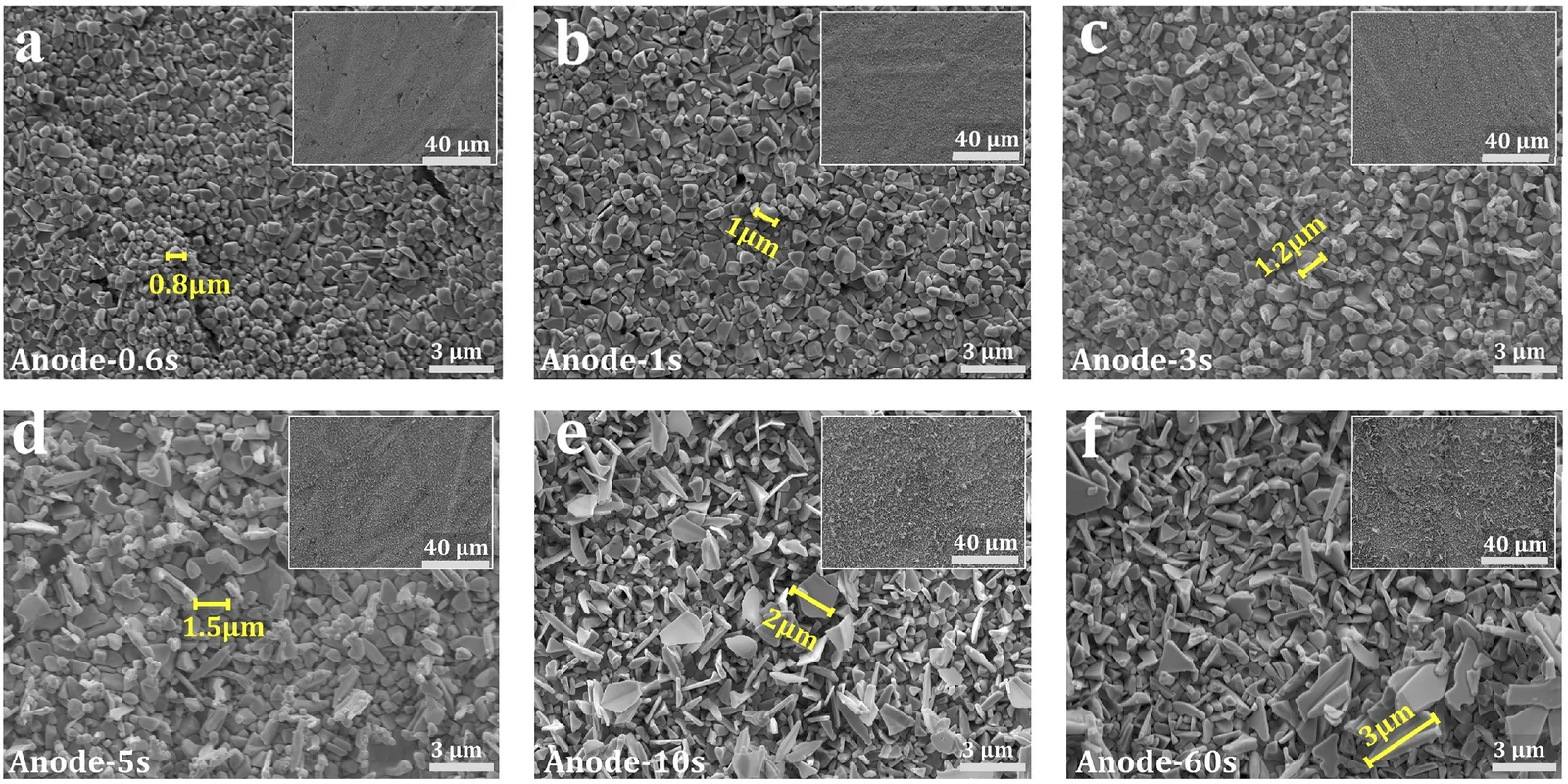
(a–f) SEM images of anodes deposited under condition B (Table S1) for various electrolysis times (as indicated).

To elucidate the formation mechanisms of white CuBr on the anode and the fern-like UCP on the cathode, this study focused on the temporal evolution of the surface microstructures on the both electrodes during early-stage electrochemical deposition. As shown in Fig. 6, the anode surface underwent a remarkable transition from smooth to rough morphology within the initial 60 s of electrolysis. The CuBr particles exhibited dynamic morphological changes, with progressive growth being observed in the first 3 s, followed by the development of small flake-like structures (∼1.5 µm) at 5 s. These flake-like particles further evolved into larger (∼3 µm) three-dimensional porous structures by 60 s. Continued electrolysis leads to increased surface roughness, accompanied by the development of a looser and more differentiated structure. Post-electrolysis scraping collected flake-like particles and granular structures, yielding a mixture of flakes and spheres (Fig. 5d).

**Fig. 7:**
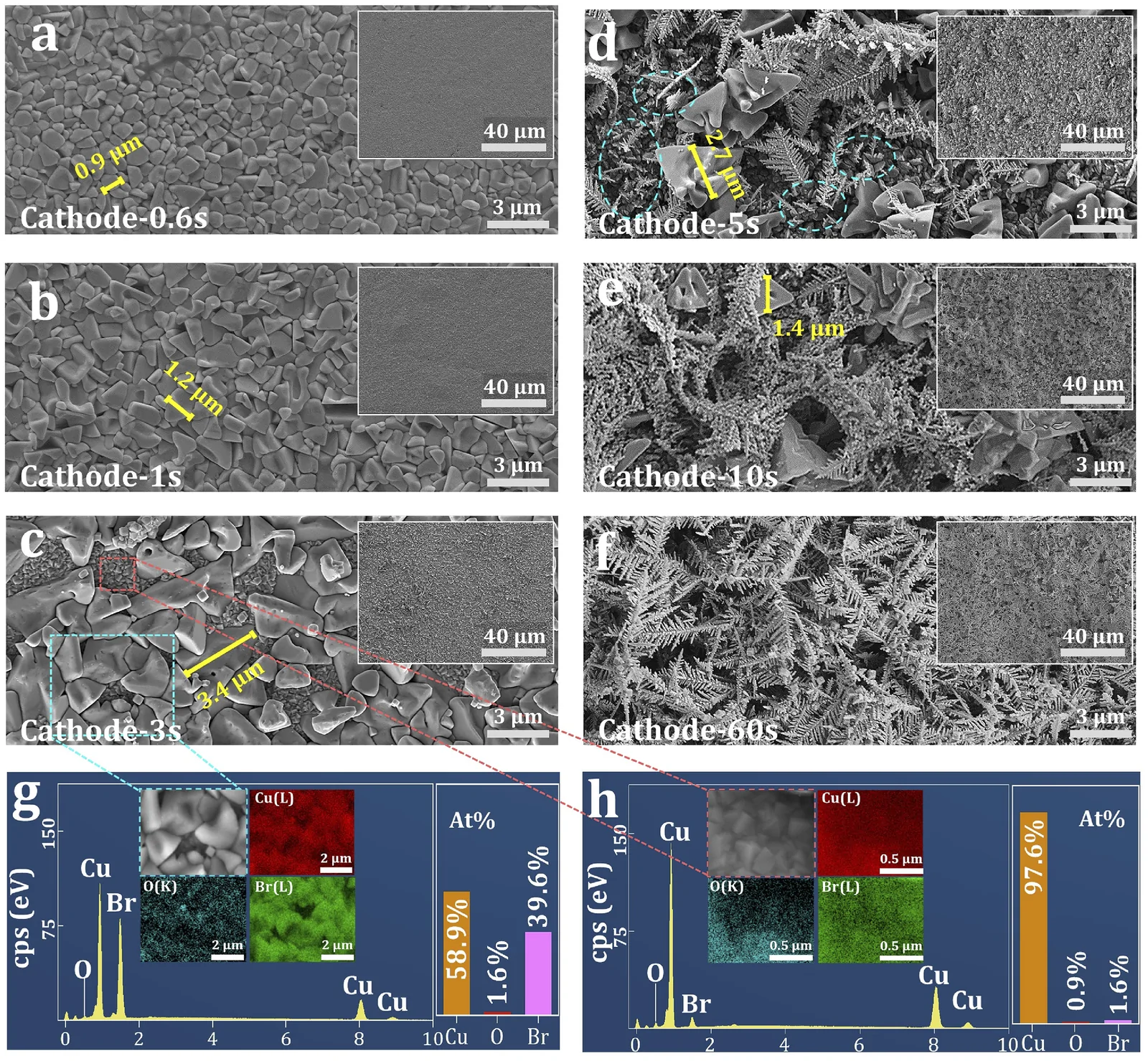
(a–f) SEM images of cathodes deposited under condition B (Table S1) for various electrolysis times (as indicated). (g and h) EDS spectra of the selected regions from image (c).

The cathodic electrodeposition followed distinct morphological evolution pathways, contrasting sharply with anodic behavior (Fig. 7a–f). Initial nucleation (≤1 s) generated geometrically ordered microparticles that evolved from ∼0.9 µm (0.6 s) to ∼3.4 µm (3 s) through sequential expansion and structural reorganization. Subsequent transformation at 3 s manifested through cavity formation and edge recession in primary particles, concurrent with the emergence of secondary microparticles (∼0.2 µm). This restructuring produced a transitional architecture in which partially collapsed Cu–Br-rich regions containing 58.9 at% Cu and 39.6 at% Br coexisted with Cu-rich particles containing 97.6 at% Cu, as indicated by EDS (Fig. 7g and h). By 5 s, the structural evolution bifurcated into two distinct pathways: (i) continued collapse of Cu–Br particles to ∼2.7 µm, and (ii) surface dominance of growing Cu microparticles (∼0.3 µm) undergoing morphological differentiation. This differentiation generated dendritic precursors (Fig. S21, cyan circles) alongside mature fractal dendrites that extend beyond the original Cu–Br framework, establishing a new transformation regime. Dendritic proliferation underwent dramatic acceleration by 10 s, with the evolving branched architecture progressively encapsulating both spherical microparticles and residual structures. The transformation reached completion at 60 s, producing an interconnected network of fern-like dendrites that established a characteristic loose, porous morphology, which ultimately obscured all initial architectural features. To evaluate whether the disappearance of the cathodic CuBr-rich deposits could be explained solely by non-electrochemical dissolution, 1 g of independently prepared γ-CuBr was immersed in 100 mL of the condition B electrolyte at 50 °C under stirring (300 rpm) for 5 min. The measured CuBr-equivalent dissolved concentration was only 0.787 mM, indicating limited solubility on the experimentally relevant timescale. In addition, under the same bulk electrolyte composition and temperature, the CuBr formed at the anode could be recovered after electrolysis and identified as crystalline γ-CuBr (Fig. 5). These control results indicate that chemical dissolution and thermal exposure alone are insufficient to account for the rapid disappearance of the cathodic CuBr-rich structures, although a partial contribution from dissolution cannot be completely excluded.

Based on the aforementioned analysis, this study investigated the reaction mechanism of Cu electrolysis in the presence of HBr. During the electrolytic process, a white CuBr layer initially forms on the cathode surface, followed by Cu deposition accompanied by significant HER, while CuBr is consistently generated at the anode. In the CuSO_4_–H_2_SO_4_–HBr ternary system, the cathodic reaction undergoes a proposed sequence involving CuBr-rich species: Cu^2+^ → Cu^+^Br-rich → Cu^0^, whereas the anodic reaction follows Cu^0^ → Cu^+^Br. The fundamental reaction steps are outlined as follows:

Cathodic reactions:

1 Cu^2+^+ e^−^ + Br^−^ → Cu^+^Br

2 Cu^+^Br + e^−^ → Cu^0^ + Br^−^

Anodic reaction:

3 Cu^0^ − e^−^ + Br^−^ → Cu^+^Br

It is noteworthy that the concentrations of Br^−^ and Cu^2+^ in the electrolyte are relatively low and rapidly deplete at the reaction interface as electrolysis proceeds. This concentration polarization consequently reactivates the intrinsic electrode reactions of the binary CuSO_4_–H_2_SO_4_ system, enabling their parallel progression:

Cathodic reactions:

4 Cu^2+^ + 2e^−^ → Cu^0^

5 2H^+^ + 2e^−^ → H_2_

Anodic reaction (copper anode):

6 Cu − 2e^−^ → Cu^2+^

**Fig. 8:**
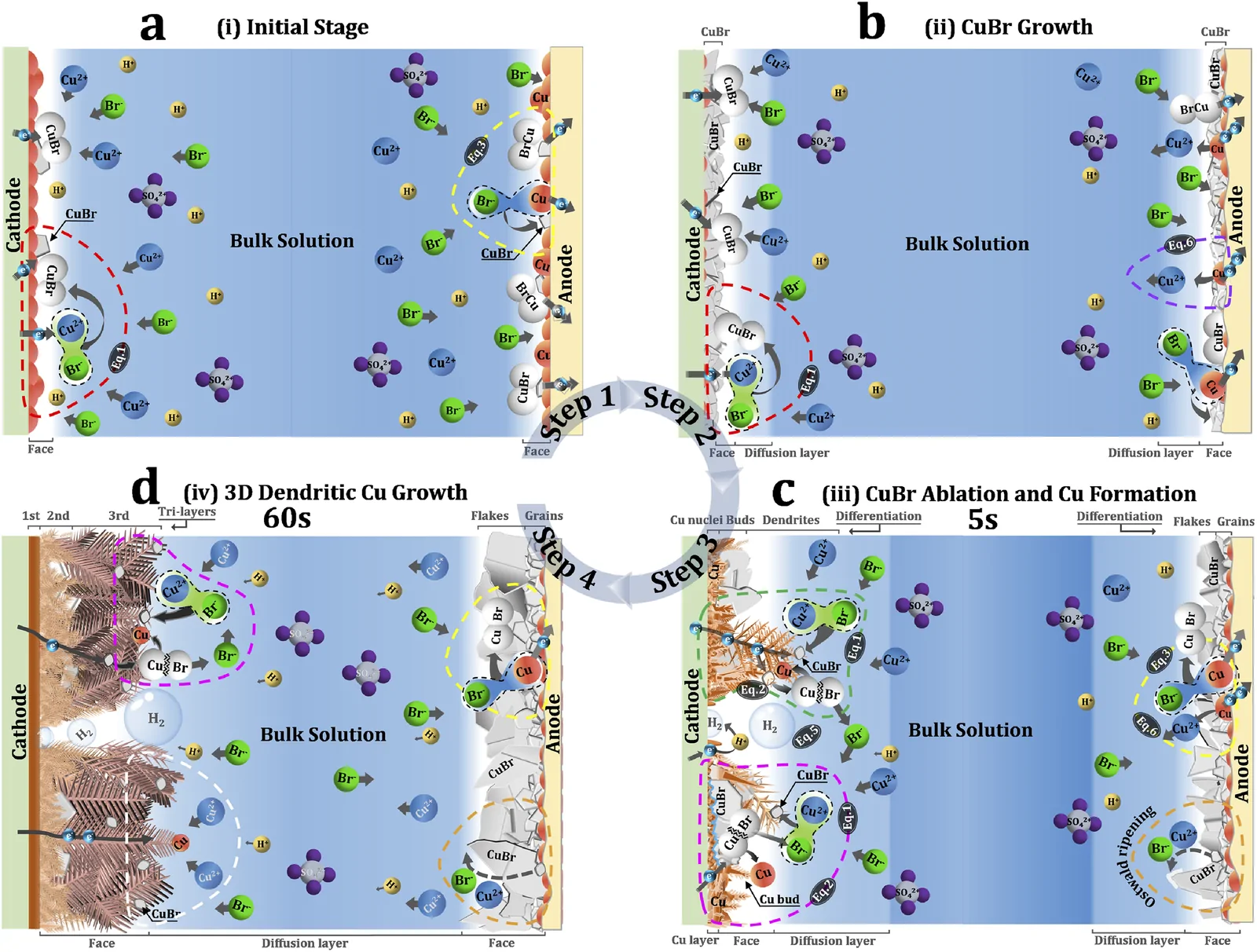
(a–d) Schematic illustration of coupled cathode–anode behaviors and associated reaction mechanisms during electrochemical synthesis of fern-like copper powders in a CuSO_4_–H_2_SO_4_–HBr electrolyte system.

Furthermore, this study proposes a time-dependent model (Fig. 8) to elucidate the electrode behaviors and reaction mechanisms underlying the synthesis of fern-like UCP. The proposed model illustrates that rapid Cu^2+^ and Br^−^ consumption at the reaction interface in the CuSO_4_–H_2_SO_4_–HBr system induces concentration polarization, shifting the reaction kinetics from interface-controlled to diffusion-limited regimes. At the cathode, CuBr nuclei transform into highly structured fern-like UCP, while the anode forms size-gradient flake-like CuBr particles. Mechanistically, Br^−^ acts as a bridge, mediating electron transfer *via* a bromide bridge mechanism, which is central to the bromide-electrocatalyzed formation of hierarchical fern-like UCP structures. The rapid depletion of Cu^2+^ and Br^−^ at the interface further enhances this process, promoting the cathodic transformation and anode flake formation. The deposition process can be divided into four key stages.

(i) Initial stage (≤1 s, Fig. 8a): Upon electrification, Cu^2+^ and Br^−^ are initially abundant near the cathode–electrolyte interface (Face). The rapid appearance of CuBr-rich deposits indicates that eqn (1) is kinetically favorable during this early interval. Although direct Cu^2+^ reduction and HER cannot be completely excluded, the earliest phase and morphological observations are dominated by CuBr-rich deposition. At the applied current density of 1500 A m^−2^, strong cathodic polarization promotes rapid formation of CuBr-rich nuclei on the cathode surface. At the anode, Cu^0^ is oxidized in the presence of Br^−^ to form CuBr particles through eqn (3), producing the characteristic anode corrosion morphology. The porous CuBr layer permits sustained transport of Br^−^ to the underlying Cu surface.

(ii) CuBr formation stage (1–2 s, Fig. 8b): a layer of CuBr particles forms at both the cathode and anode surfaces. Under high current density, the rapid consumption of Br^−^ near the both Faces and Cu^2+^ near the cathode Face significantly reduces their concentrations, establishing a pronounced concentration gradient. This gradient drives the transformation of initially formed small CuBr particles into enlarged flake structures, causing surface roughening. Simultaneously, the anode may initiate reaction eqn (6) (purple circles), releasing Cu^2+^ ions at the anode Face. As the reaction progresses, the Faces of both electrodes migrate toward the electrolyte center, forming distinct diffusion layers (as indicated).

(iii) CuBr ablation and Cu formation stage (2–5 s, Fig. 8c). Concentration polarization becomes severe, depleting Cu^2+^ and Br^−^ near the cathode surface to near zero and thereby suppressing further CuBr formation.^43^ Under these conditions, HER (eqn (5)), direct Cu^2+^ reduction (eqn (4)), and the proposed electrochemical consumption of CuBr-rich species (eqn (2)) proceed concurrently. At the substrate-CuBr interface, the CuBr-rich deposits in direct contact with the conductive substrate may undergo electrochemical consumption under continued cathodic polarization, accompanied by the emergence of numerous fine Cu-rich nuclei. As the reaction proceeds, this interface gradually evolves into a substrate-supported dense Cu layer. Electrons conduct through this dense Cu layer to the Cu-CuBr interface (cyan lines in pink circles, Fig. 8c), where the CuBr-rich layer progressively recedes while Cu-rich particles emerge and grow in adjacent regions. The temporally correlated disappearance of CuBr-rich structures and formation of Cu-rich particles is consistent with, but does not directly prove, the proposed electrochemical reduction of CuBr to metallic Cu. Chemical dissolution and mechanical detachment may also contribute to the depletion of the CuBr-rich deposits. The deposited Cu layer subsequently develops outward, exhibiting continuous inward-to-outward growth of Cu-rich nuclei. As the early CuBr-rich deposits become substantially depleted, the rapid proliferation of Cu nuclei decreases, and their further growth increasingly relies on the supply of Cu^2+^ from the bulk electrolyte. This re-establishes concentration polarization, promoting outer-layer grain differentiation and growth. Larger grains develop bud-like Cu structures, with a few evolving into sporadic dendritic morphologies. These 3D dendrites extend beyond the concentration polarization zone, accessing Cu^2+^ and Br^−^ from the bulk electrolyte, where new CuBr-rich deposits may form locally at the dendrite tips through eqn (1) (green circles in Fig. 8). These locally formed CuBr-rich species may subsequently be electrochemically consumed, while direct Cu^2+^ reduction proceeds in parallel, contributing to further Cu deposition and fractal dendritic growth. Concurrently at the anode, significant Br^−^ consumption near the anode surface causes CuBr growth to diverge into two modes (Fig. 6): (1) inward growth, where eqn (3) is suppressed due to Br^−^ depletion and eqn (6) proceeds in parallel, leading to continued anode thinning and slow inward CuBr growth once a stable Br^−^ diffusion layer is established; and (2) outer-layer evolution, where CuBr particles distant from the anode experience growth stagnation due to metallic Cu (Cu^0^) depletion. In this region, the observed reduction in the number of fine CuBr particles and concurrent growth of larger particles may be associated with surface-energy-driven coarsening or Ostwald ripening. Although this interpretation is consistent with the time-dependent morphological observations, the detailed coarsening mechanism has not been directly established.

(iv) 3D dendritic Cu growth stage (>5 s, Fig. 8d): as the diffusion layer stabilizes, Cu growth at the cathode reaches a stable and mature stage, forming a well-defined tri-layer structure (Fig. S8): (1) a thin, dense 2D layer (1st layer) tightly bonded to the substrate; (2) an outer layer (3rd layer) of loosely interwoven fern-like 3D dendrites with highly developed fractal features, exhibiting complex secondary and tertiary branches that form towering, luxuriant morphologies accompanied by HER-induced voids; and (3) an intermediate layer (2nd layer) of relatively loose, underdeveloped fine Cu dendrites interspersed with a few coarse CuBr particles. Shielded by the outer dendritic layer and constrained by limited growth space and reactant supply, the intermediate layer expands slowly. During electrolysis, both the intermediate and outer layers continue to extend, with the outer dendrites growing significantly faster due to preferential access to reactants. Upon completion, the loose 3D dendrites in the outer layer form powder upon scraping, while the dense inner layer resists physical removal, remaining on the cathode surface.

In industrial applications, maintaining Cu^2+^ concentration below 0.1 M and optimizing current density in the range of 1200–1800 A m^−2^ achieves a comprehensive optimization of apparent density, particle size, current efficiency, and energy consumption.^18^ Under typical UCP production conditions (condition D, Table S1), a current density of 1500 A m^−2^ is ∼5 times the limiting current density.^44^ This combination of high current density and low Cu^2+^ concentration lowers the nucleation barrier, enhances Cu^2+^ reduction rates, and promotes the generation of numerous nuclei and grain refinement,^45^ resulting in a dense deposition layer (Fig. S4–S8). The initial deposition follows an electroplating mechanism, forming a dense 2D Cu structure that grows according to Faraday's law and exhibits a planar growth mode in the initial ECP stage.^46,47^ Additionally, the reduced Cu forms interlocking metallic bonds with the Cu substrate, enabling seamless adhesion of the dense deposition layer, likely due to direct interactions between the initially reduced Cu and the substrate, as well as crystallographic matching.

### 2.4 Optimization of bromide concentration and multifunctional performance of fractal UCP

The influence of Br^−^ concentration (0–600 mM) on UCP characteristics was systematically investigated under condition C (Table S1). The synthesized copper powders were designated as P-*x*Br, where *x* represents the Br^−^ concentration in mM.

**Fig. 9:**
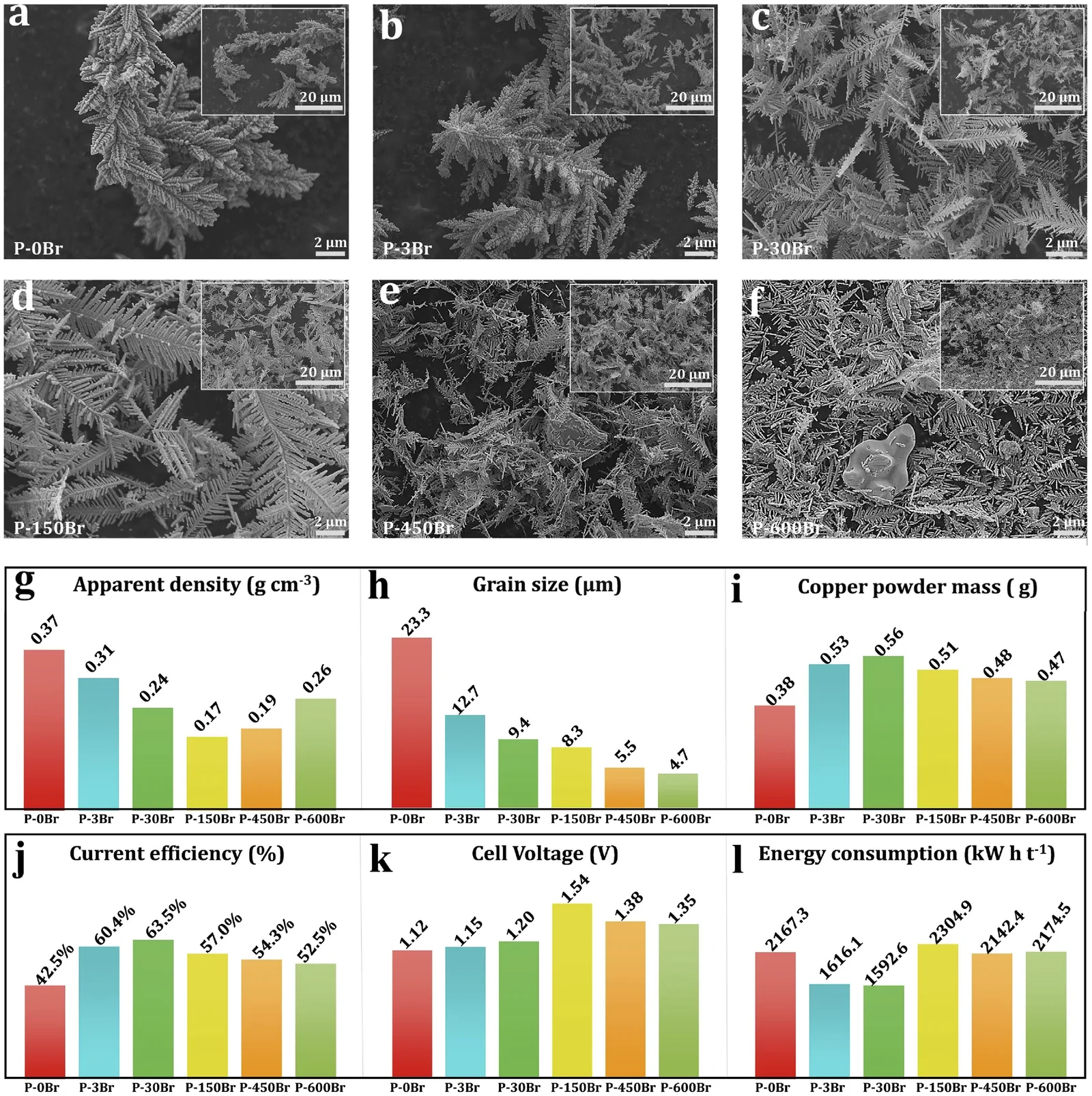
(a–f) SEM images, (g) apparent density, (h) grain size, (i) powder mass, (j) current efficiency, (k) cell voltage, and (l) energy consumption of the copper powders deposited from the electrolytes with varying Br^−^ concentrations (as indicated) under condition C (Table S1).

SEM analysis (Fig. 9a–f) revealed a concentration-dependent morphological evolution. The Br^−^-free sample (P-0Br) exhibited bulky, aggregated sorghum-like particles with disordered branching. Introduction of 3 mM Br^−^ (P-3Br) induced a transition to fractal dendritic structures with reduced particle size. At 30 mM Br^−^, well-defined main trunks and highly ordered fern-like dendrites emerged, while 150 mM Br^−^ yielded the highest degree of fractal complexity. Excessive Br^−^ concentrations (≥450 mM) caused dendritic shrinkage, fragmentation, and the formation of blocky Cu crystals (Fig. 9e, f and S22). Beyond morphology, Br^−^ incorporation also significantly enhanced UCP performance (Fig. 9g and h). The introduction of Br^−^ (3–150 mM) reduced apparent density (0.31 → 0.17 g cm^−3^) and particle size (12.7 → 8.3 µm). Higher concentrations (450–600 mM) increased apparent density (0.19–0.26 g cm^−3^) while further reducing the size (5.5–4.7 µm) due to dendritic shrinkage. The maximum recovered powder mass and current efficiency were obtained at 30 mM Br^−^. Under condition C, the specific direct-current energy consumption decreased from 2167.3 kWh t^−1^ for the Br^−^-free control to 1592.6 kWh t^−1^ at 30 mM Br^−^, corresponding to a 26.5% reduction (Fig. 9i–l). Increasing the Br^−^ concentration to 150 mM promoted the formation of a white CuBr-rich layer on the anode, which increased the cell voltage and resulted in a 44.7% increase in specific direct-current energy consumption relative to the value obtained at 30 mM Br^−^. At Br^−^ concentrations of 300–600 mM, spontaneous detachment of the cathodic deposit partially offset the increase in specific direct-current energy consumption associated with the elevated cell voltage. These findings demonstrate that an appropriate Br^−^ concentration promotes copper deposition and dendritic branching while improving the physical characteristics and electrolysis performance of UCP. Precise control of Br^−^ concentration therefore enables the preparation of UCP with tunable particle size, apparent density, and dendritic morphology.

In industrial copper powder production, cathodic cyclic deposition is extensively employed to optimize production efficiency and minimize costs. Maintaining cathode uniformity and surface smoothness is crucial for ensuring the stability and efficiency of prolonged electrolysis. This study systematically investigated the impact of Br^−^ concentration (0–600 mM) on the weight and surface characteristics of copper cathodes in a ternary electrolyte system (1.53 M H_2_SO_4_, 0.08 M CuSO_4_, and *x* mM HBr, where *x* = 0–600) during 180 s electrolysis. Post-electrolysis, cathodes were subjected to brushing, cleaning, and drying procedures, with samples labeled as C-*x*Br based on Br^−^ concentration, with the pristine cathode designated as C-pristine. The experimental results demonstrate the critical role of Br^−^ concentration in cathode surface properties. Br^−^-free conditions (C-0Br) resulted in substantial residual copper deposits, yielding a rough surface texture (Fig. S23a) that resisted removal through brushing. This surface roughness, quantified by a 5.8-fold rise (Fig. S23b), was accompanied by a 61 mg weight gain (Fig. S23c) and enhanced oxidation susceptibility, as evidenced by surface darkening (Fig. S23a). Optimal Br^−^ concentrations (3–30 mM) effectively eliminated cathode residues while preserving surface smoothness, thereby enhancing cathode recyclability and ensuring consistent product quality and production efficiency. However, excessive Br^−^ concentrations (≥150 mM) detrimentally affected cathode surface properties, with both residual mass and surface roughness exhibiting significant concentration-dependent increases.

**Fig. 10:**
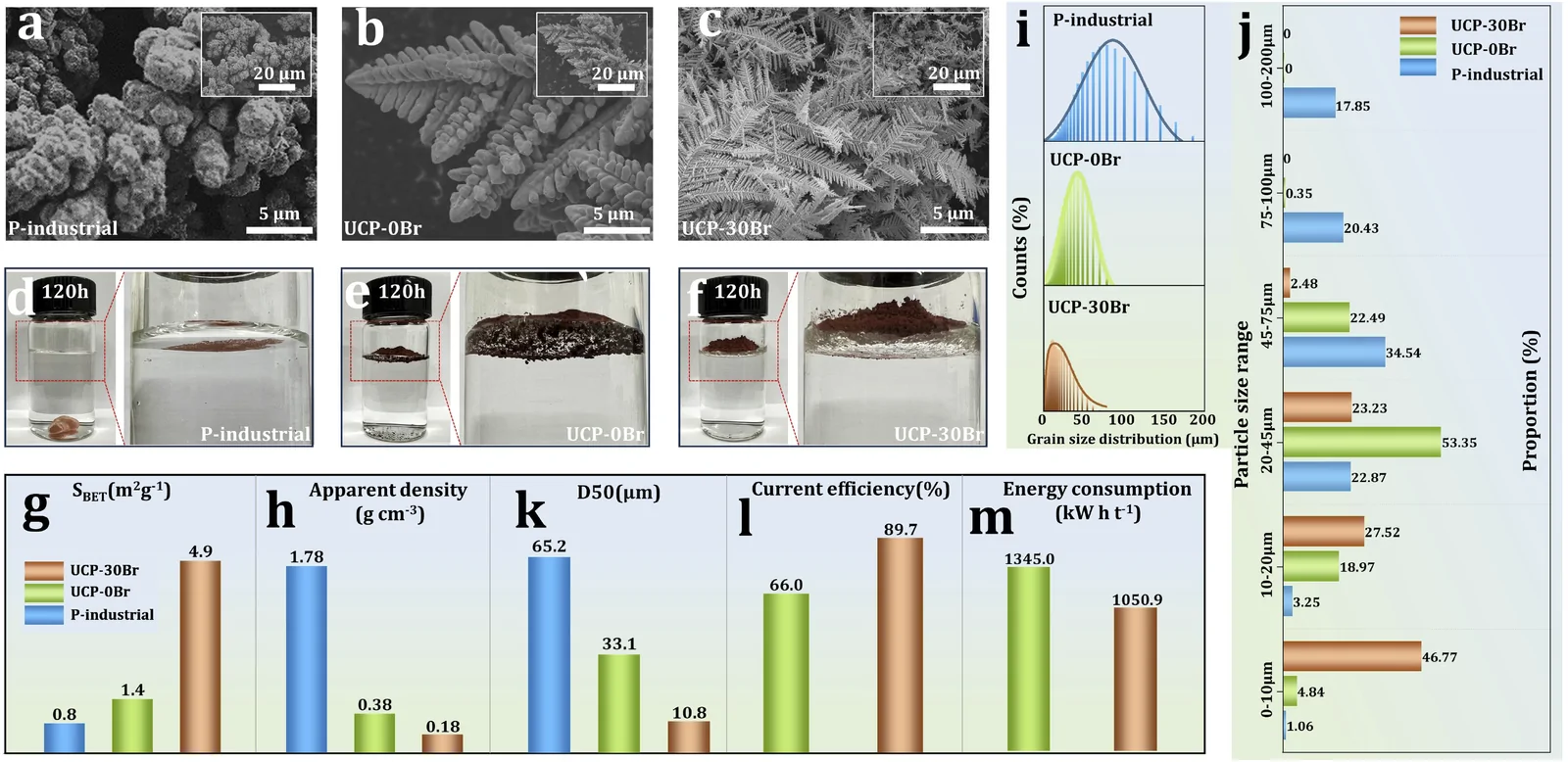
SEM images of (a) industrial copper powder (P-industrial) obtained from a factory, and copper powders deposited (b) without Br^−^ (UCP-0Br) and (c) with 30 mM Br^−^ (UCP-30Br) under condition D (Table S1). (d–f) Photographs of 0.5 g P-industrial, UCP-0Br, and UCP-30Br powders (as indicated) after a 120 h free-settlement test. Performance characteristics of these copper powders: (g) BET specific surface area (*S*_BET_), (h) apparent density, (i) particle size distributions with (j) related numerical analysis (k) median particle size (D50), (l) current efficiency, and (m) energy consumption.

To evaluate the performance of Br^−^-modified UCP synthesis under production-relevant laboratory conditions, comparative experiments were conducted under condition D (Table S1). UCP samples were prepared for 60 min in the absence of Br^−^ (UCP-0Br) and in the presence of 30 mM Br^−^ (UCP-30Br). A commercial industrial copper powder (P-industrial) was included as a reference. Morphological analysis revealed distinct structural features among the three powders (Fig. 10a–c). P-industrial exhibited bulky, short-branched sorghum-like aggregates, whereas UCP-0Br developed more elaborate and ordered dendritic structures. UCP-30Br exhibited a finely developed fern-like morphology with clearly defined main trunks, regularly distributed secondary branches, and a pronounced hierarchical fractal architecture. A 120 h free-settling experiment (Fig. 10d–f) further demonstrated the differences in the physical behavior of these powders in water. P-industrial settled completely, whereas UCP-0Br formed a partially suspended surface layer. In contrast, UCP-30Br remained predominantly at the water surface and formed a relatively thick and persistent floating powder layer. This behavior is qualitatively consistent with limited wetting and enhanced air retention in the hierarchical UCP-30Br powder. Brunauer–Emmett–Teller analysis (Fig. 10g) showed that UCP-30Br had a specific surface area of 4.9 m^2^ g^−1^, which was 6.1 times that of P-industrial and 3.5 times that of UCP-0Br. UCP-30Br also exhibited an ultralow apparent density of 0.18 g cm^−3^ (Fig. 10h). Particle-size distribution analysis (Fig. 10i–k) showed that P-industrial had a broad size range of 0–186 µm, with the largest fraction occurring between 40 and 80 µm and only 1.06% of the particles smaller than 10 µm. UCP-0Br exhibited a narrower distribution of 0–87 µm, with the largest fraction between 20 and 40 µm. UCP-30Br showed a further narrowed distribution of 0–70 µm, with 46.77% of the particles smaller than 10 µm, a D50 of 10.8 µm, and a D90 below 45 µm. Under condition D, UCP-30Br achieved a current efficiency of 89.7% and a specific DC energy consumption of 1050.9 kWh t^−1^, representing a 21.9% reduction relative to UCP-0Br (Fig. 10l and m). Composition analysis (Fig. S24) further showed that UCP-30Br consisted predominantly of Cu (95.51 at%), with minor surface oxygen (4.46 at%) and negligible residual Br (0.02 at%). Together, these results demonstrate that Br^−^-modified electrodeposition enables the preparation of UCP with a highly developed fractal dendritic architecture, an ultralow apparent density, a high specific surface area, and favorable laboratory electrolysis performance. These characteristics make UCP-30Br a promising candidate for antimicrobial applications and powder-based interfaces requiring controlled wetting behavior, and suggest potential utility in electrochemical and electronic material systems.

**Fig. 11:**
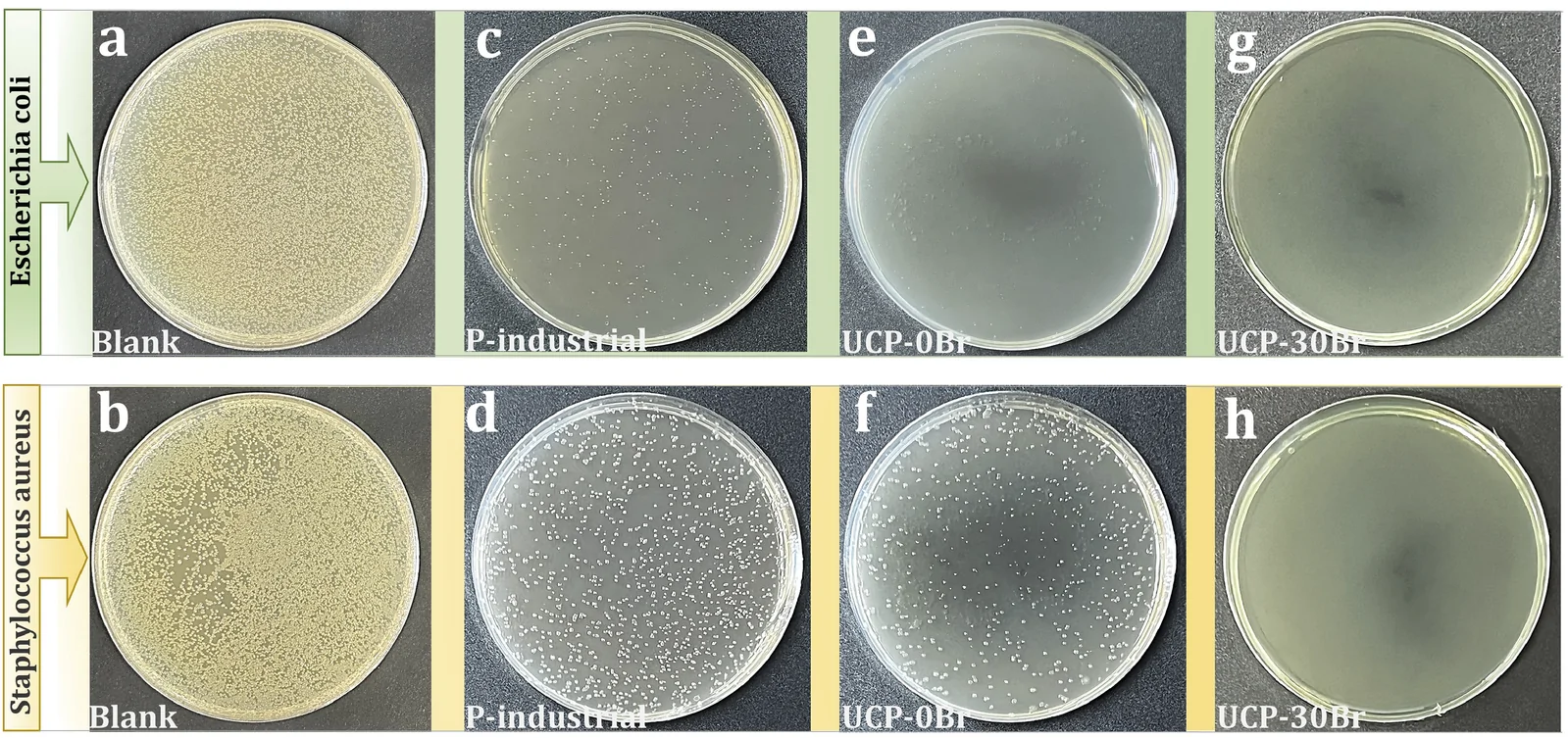
Macrographs comparing the colony counts of *Staphylococcus aureus* and *Escherichia coli* after 12 h co-culture with various copper powder liquid antibacterial agents: (a and b) blank control group, (c and d) P-industrial, (e and f) UCP-0Br, and (g and h) UCP-30Br.

This study explored the multifunctional potential of fractal fern-like UCP synthesized *via* bromide assistance, focusing on its antibacterial activity and the wetting behavior of standardized powder beds. Antibacterial performance was evaluated against *Escherichia coli* (*E. coli*) and *Staphylococcus aureus* (*S. aureus*). Bacterial suspensions were exposed to the different copper powders for 12 h, and the number of culturable bacteria was determined using the plate-counting method. PBS containing the bacterial suspension but no copper powder served as the untreated control. P-industrial, UCP-0Br, and UCP-30Br were tested at a powder concentration of 0.50 g L^−1^. As shown in Fig. 11, all three copper powders reduced the number of culturable bacteria, although their antibacterial efficiencies differed substantially. P-industrial produced colony-count reductions of 95.1% for *E. coli* and 88.4% for *S. aureus*, whereas UCP-0Br produced reductions of 98.6% and 94.1%, respectively. Following treatment with UCP-30Br at 0.50 g L^−1^ for 12 h, no culturable colonies were detected of either bacterial strain were detected within the detection limit of the plate-counting assay. To assess the concentration dependence, UCP-30Br was tested at 0.05, 0.20, and 0.50 g L^−1^ (Fig. S25). Each condition was tested using the same bacterial inoculation, 12 h exposure, plating, and colony-counting procedures. For *E. coli*, UCP-30Br produced relative colony-count reductions of 87.3% and 95.3% at 0.05 and 0.20 g L^−1^, respectively, while no viable colonies were detected at 0.50 g L^−1^. For *S. aureus*, the corresponding reductions were 93.4% and 97.2% at 0.05 and 0.20 g L^−1^, respectively, and no viable colonies were detected following treatment at 0.50 g L^−1^. The corresponding agar-plate and SEM images are presented in Fig. S26. These results demonstrate a clear concentration-dependent antibacterial effect.

The antibacterial activity of copper-based materials is generally associated with coupled contact- and ion-mediated mechanisms.^48–51^ Upon contact with bacterial cells, copper particles can adsorb onto bacterial surfaces through electrostatic interactions, disturb the integrity of the cell wall and membrane, and promote local oxidative stress, which may subsequently cause damage to intracellular components, including proteins, lipids, and DNA.^52^ In parallel, copper ions released from the particle surface, particularly Cu^2+^ species, can disrupt intracellular redox homeostasis and accelerate oxidative damage to biomolecules, thereby further enhancing antibacterial efficacy.^53,54^ The superior antibacterial performance of UCP-30Br under the present assay conditions can be attributed to a synergistic combination of multiple factors. First, its highly developed fern-like fractal architecture with a large specific surface area of 4.9 m^2^ g^−1^ (Fig. 10g) exposes abundant active sites and numerous sharp micro-dendritic protrusions, which enhance physical contact with bacterial cells. Second, the enhanced interfacial interactions arising from this hierarchical morphology may promote more effective disruption of bacterial cell integrity. Third, the elevated level of soluble copper species released from UCP-30Br, as confirmed by ICP-MS (Table S3), likely contributes to the overall antibacterial effect. Therefore, the pronounced antibacterial effect observed for UCP-30Br at 0.50 g L^−1^ after 12 h is reasonably attributed to a synergistic combination of multiple factors, including enhanced physical contact enabled by the fractal architecture, increased interfacial interactions, and elevated release of soluble copper species, although the present data do not allow these contributions to be distinguished or individually quantified.

**Fig. 12:**
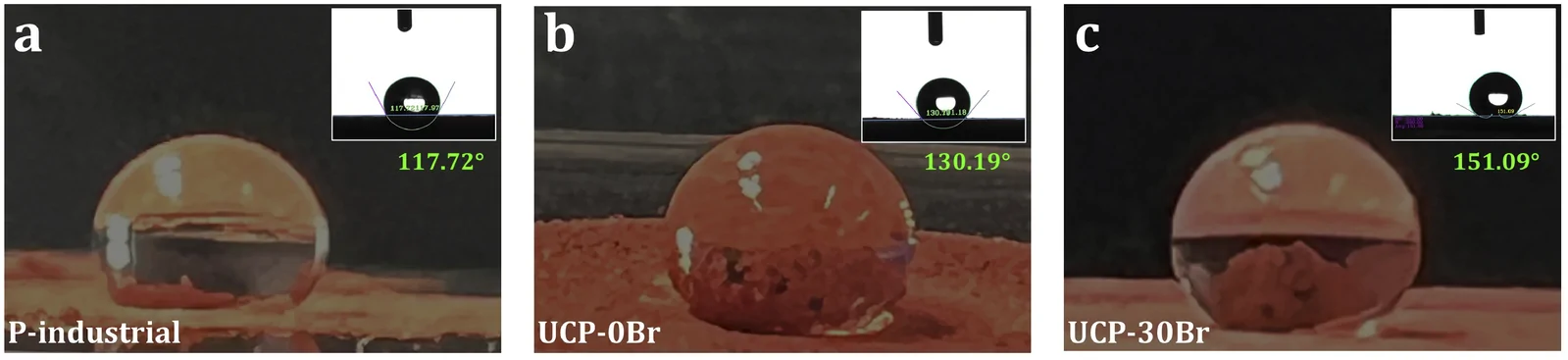
Droplet morphology on standardized vibration-leveled powder beds of (a) P-industrial, (b) UCP-0Br, and (c) UCP-30Br. The insets show the static contact angles.

To evaluate the hydrophobic properties, 1.0 g of the copper powder was vacuum-dried at 318 K for 12 h. Then, 0.2 g of the dried powder was transferred to a sample dish (50 mm × 35 mm × 2 mm) and compacted through vibration to form a flat surface. A 3 µL droplet of deionized water was deposited onto the surface using a microsyringe, and static images of the droplet morphology were captured in a natural environment. As shown in Fig. 12, distinct droplet morphologies were observed on the standardized powder beds. The apparent static water contact angle increased from 117.72° for P-industrial to 130.19° for UCP-0Br and 151.09° for UCP-30Br. These results demonstrate progressively higher apparent static water contact angles and lower apparent wettability of the prepared powder beds, with UCP-30Br exhibiting the highest value. The higher apparent static water contact angle of UCP-30Br can be attributed to its hierarchical micro-/nanoscale dendritic morphology, which increases surface roughness and facilitates air retention within interparticle and interdendritic voids. This interpretation is qualitatively consistent with the Cassie–Baxter framework.^55–57^ In this state, the droplet sits on a composite surface of solid peaks and trapped air pockets, drastically reducing the solid–liquid contact area. The intricate network formed by the interwoven hierarchical primary and secondary dendrites and their deep crevices in UCP-30Br (Fig. 10) provides an ideal condition for facilitating this air-trapping effect.

## 3 Conclusions

This study demonstrates that bromide-induced modulation of copper deposition provides an effective strategy for combining ultralow apparent density and well-developed fractal architectures with high current efficiency and reduced energy consumption during UCP synthesis. Systematic evaluation of four electrolyte additives identified Br^−^ as an effective regulator of copper electrodeposition. Electrochemical measurements, potential-dependent phase identification, and time-resolved *ex situ* characterization support the transient participation of CuBr-rich species and the emergence of an additional CuBr-mediated reduction route alongside direct Cu^2+^ reduction. Based on the observed temporal evolution, a four-stage growth model is proposed, involving (i) formation and assembly of CuBr-rich particles, (ii) progressive disappearance and structural collapse of the early deposits under continued cathodic polarization, (iii) emergence and fractal reconstruction of Cu-rich particles, and (iv) diffusion-dominated growth of fern-like dendrites. Under the optimized condition with 30 mM Br^−^, UCP-30Br exhibited an ultralow apparent density of 0.18 g cm^−3^, a specific surface area of 4.9 m^2^ g^−1^, a narrow particle-size distribution (D50 of 10.8 µm, with 46.77% of particles below 10 µm), and a high apparent static water contact angle of ∼151° on a standardized powder bed. The Br^−^-modified process achieved a current efficiency of 89.7% and a specific energy consumption of 1050.9 kWh t^−1^, representing a 21.9% reduction relative to the Br^−^-free control. Under the tested conditions, UCP-30Br at 0.50 g L^−1^ reduced culturable Gram-positive and Gram-negative bacteria to below the detection limit after 12 h of exposure. These functional properties are attributed to the highly developed hierarchical architecture and high specific surface area, which enhance physical contact with bacterial cells, increase interfacial interactions, elevate the release of soluble copper species, and promote air trapping within the multiscale surface structure. Overall, these results establish a promising bromide-modulated strategy for the energy-efficient synthesis of hierarchical UCP and offer mechanistic insights for the structural design of electrodeposited metallic materials.

## Author contributions

Keshu Song, Youpo Mise, Shaohua Wang, Yakun Yin and Wenqiang Yang carried out the experiments; Wenqiang Yang conceived the project; Wenqiang Yang, Xiaoli Yuan, Wentang Xia, Xuejiao Zhou and Juan An supervised the electrochemical cell and the electrochemical aspects of the project; and all authors cowrote the manuscript.

## Conflicts of interest

On behalf of all authors, the corresponding author states that there is no conflict of interest.

## Supplementary Material

RA-016-D6RA04989C-s001

RA-016-D6RA04989C-s002

RA-016-D6RA04989C-s003

## Data Availability

The data that support the findings of this study are available from the corresponding author upon reasonable request. Supplementary information (SI) is available. See DOI: https://doi.org/10.1039/d6ra04989c.
