# Supplementary material for: Bromide-mediated pathway modulation enables energy-efficient electrosynthesis of ultralight hierarchical copper powder
Source: RSC Adv. 2026 Aug 21;16(41):49269–85. doi: 10.1039/d6ra04989c (PMC13497336; doi:10.1039/d6ra04989c)
Supplement: Supplementary file 1 [file RA-016-D6RA04989C-s001.pdf]

Supplementary Information (SI) for RSC Advances.
This journal is © The Royal Society of Chemistry 2026

Supporting Materials

Bromide-Mediated Pathway Modulation Enables Energy-Efficient Electrosynthesis of Ultralight Hierarchical Copper Powder

Keshu Song,[a] Youpo Mise,[a] Shaohua Wang,[a] Yakun Yin,[a] Juan An,[a,b] Xuejiao Zhou,[a,b] Xiaoli Yuan,[a,b] Wentang Xia,[a,b] Wenqiang Yang,[a,b,*]

a School of Metallurgy and Power Engineering, Chongqing University of Science and Technology, Chongqing 401331, P.R. China

b Chongqing Municipal Key Laboratory of Institutions of Higher Education for Value-added Treatment and Green Extraction from Complicated Resources, Chongqing 401331, P.R. China

[*] Corresponding author. Tel: +86-023-65023711; Fax: +86-023-65023711.

E-mail address: wenqiangyang@cqust.edu.cn (W.Q. Yang)

1. Experimental section

1.1 Chemicals and reagents

Copper sulfate pentahydrate ($CuSO_4·5H_2O$, ≥99%), hydrobromic acid (HBr, 40 wt.%), sodium acetate trihydrate ($CH_3COONa·3H_2O$, ≥99%), sodium chloride (NaCl, ≥99%), potassium thiocyanate (KSCN, ≥99%), citric acid ($C_6H_8O_7$, Cit, ≥99%), potassium iodide (KI, ≥99%), acetic acid (CH_3COOH, HAc, ≥99%), ammonium bifluoride (NH_4HF_2, ≥99%), agar powder (BR), sodium thiosulfate standard solution ($Na_2S_2O_3$, 0.2 M), ethanol (C_2H_5OH), glycine (CH_2NH_2COOH, Gly, ≥99%), benzotriazole ($C_6H_5N_3$, ≥99%), and sodium hydroxide standard solution (NaOH, 0.5 M) were all procured from Aladdin Reagent (Shanghai) Co., Ltd. Sulfuric acid (H_2SO_4, ≥98%) was obtained from Chuandong Chemical Group Co., Ltd. Yeast extract (fermentation grade, BR) and peptone (BR) were supplied by Beijing Aoboxing Biotechnology Co., Ltd. Methyl red-bromocresol green indicator solution was provided by Guangzhou Testing Technology Co., Ltd. High-purity copper plates and sheets (≥99.99%) were sourced from Yunnan Copper Co., Ltd. All chemicals and reagents utilized in this study were employed without further drying or purification. Deionized water (resistivity: 18.25 $M\Omega·cm^{-1}$) for experimental use was prepared using a MOLUP-100 central ultrapure water system (MOLUP Water Co., China).

1.2 Electrolytic copper powder preparation

The experimental setup for the electrolytic copper powder preparation is shown in Fig. S1. All electrolysis experiments were conducted in a transparent polymethyl methacrylate electrolysis cell with a capacity of ~10 L (17.2 cm × 18.8 cm × 29.7 cm). The cell was connected to a 20 L reservoir, and the electrolyte was circulated through a "bottom-inlet, top-outlet" configuration. Both the cathode and anode were made of high-purity copper (≥99.99%) with an effective surface area of 100 mm × 100 mm, and were positioned with a horizontal inter-electrode distance of 20 mm. A KXN−3030D DC power supply unit (ZHAOXIN, China) was employed to deliver a constant current throughout the experiments. The electrolyte was prepared by dissolving sulfuric acid and copper sulfate pentahydrate in deionized water to yield a solution containing 0.08 M $CuSO_4$ and 1.53 M H_2SO_4. The solution was thoroughly mixed and allowed to stabilize for 24 h prior to use. The copper ion concentration in the electrolyte was determined using an indirect iodometric titration method with soluble starch as the indicator. The sulfuric acid concentration was measured by titration using methyl red-bromocresol green indicator and standardized sodium hydroxide solution. Prior to electrolysis, the cathode and anode were polished with 500, 800, and 1000-grit abrasive paper, followed by ultrasonic cleaning in

anhydrous ethanol for 30 min. The electrodes were then rinsed with deionized water, immersed in 1.53 M sulfuric acid solution for 10 min, and finally rinsed again with deionized water before being mounted in the electrolysis cell.

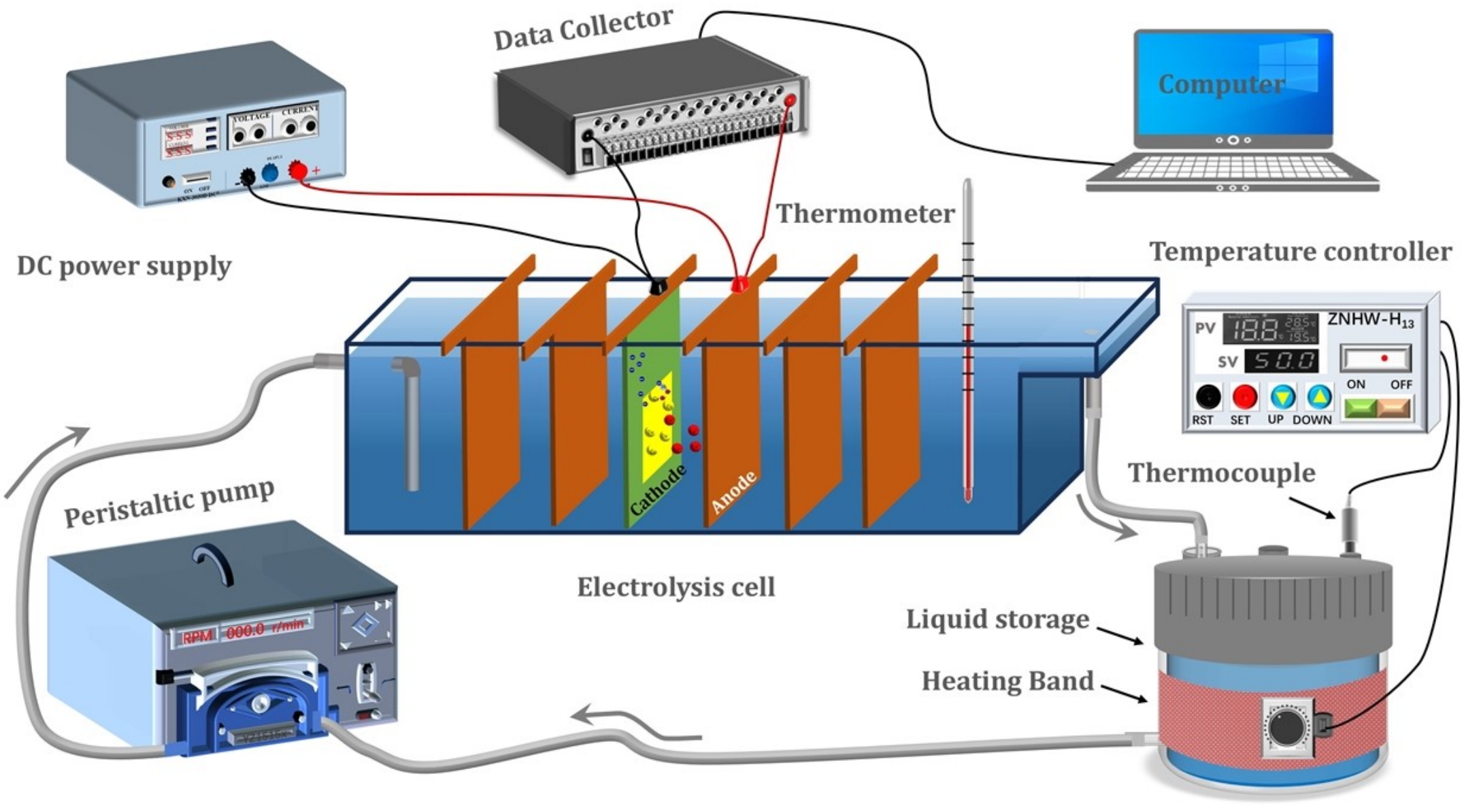

Fig. S1. The schematic diagram of experimental facility for electrolytic copper powder

After the experimental setup was completed and the parameters were adjusted, electrolysis was carried out for the predetermined duration. Upon completion, the power supply was switched off, and the cathode was carefully removed. The copper powder deposited on the cathode was transferred to deionized water and repeatedly washed until no precipitate was observed upon the addition of barium chloride solution (25 wt.%). The powder was subsequently immersed in a 0.42 mM benzotriazole (BTA) solution for 15 min to inhibit oxidation. Finally, the copper powder was dehydrated and dried in a vacuum oven at 353 K for 12 h to yield the final product. For comparison, the industrial copper powder used in this study was collected from multiple cathode plates across various electrolytic tanks at Yutai Copper Co., Ltd. (China) and underwent identical subsequent treatment processes. A comprehensive summary of the operational conditions for this study is presented in Table S1, where the symbol X denotes various additives, including citric acid (Cit), glycine (Gly), hydrobromic acid (HBr), and acetic acid (HAc).

Table S1 The deposition conditions for ECP involved in this work

Condition	$CuSO_4$ (M)	H_2SO_4 (M)	Additive (M)	Current density (A m^{-2})	Temperature (K)	Flow rate (L min^{-1})	Duration time (min)
A	0.08	1.53	X (0.15)	-1500	323	1.1	0.5 or 3
B	0.08	1.53	HBr (0.15)	-1500	323	1.1	0~3

C	0.08	1.53	HBr (0~0.60)	-1500	323	1.1	3
D	0.08	1.53	HBr (0.03)	-1500	323	1.1	60

1.3 Physical Characterization

Field emission scanning electron microscopy (FESEM) analysis was performed using a JEOL JSM-7800F instrument (JEOL Ltd., Japan), equipped with an Oxford XMax100 energy-dispersive X-ray (EDS) spectrometer, operating at an accelerating voltage of 15 keV. Particle size and distribution were measured using a BT-9300ST laser diffraction particle size analyzer (BAITE Instruments, China). The apparent density, defined as the mass per unit volume of loosely packed powder, was determined according to the GB/T 1479.3-2017 standard (Chinese National Standard for the Determination of Apparent Density of Metal Powders), using an XF-02 Hall flowmeter (JINSHISU, China). Cell voltage data were collected at a rate of one sample per second using a TP1608P–AI–A data acquisition system (Toprie, China) for subsequent computer analysis. Current efficiency and energy consumption were calculated following the method described in our previous study [1,2]. The properties of the electrolyte were analyzed using a T9 UV-Vis spectrophotometer (Purkinje, China). The specific surface area was determined from nitrogen adsorption-desorption isotherms obtained using an ASAP 2460 instrument (Micromeritics Instrument Corp., USA), employing the Brunauer–Emmett–Teller (BET) method. Surface roughness of the cathode was evaluated using a TR100 stylus-type surface roughness tester (Changzhou Sanfeng Instrument Technology Co., Ltd., China). The measurements were taken at five distinct locations on the cathode surface (as shown in Fig. S2), and the average roughness value was calculated. Nitrogen and oxygen content were determined using a HORIBA EMGA-830 oxygen/nitrogen/hydrogen (O/N/H) analyzer.

1.4 Electrochemical Analysis, Surface Wettability, and Antimicrobial Activity Assessment

All electrochemical measurements were performed using a PARSTAT 4000 (AMETEK, USA) electrochemical workstation in a three-electrode configuration. The working electrode comprised a 10 mm × 10 mm copper foil, which was polished with high-purity alumina powder (100 nm, 99.99%) and rinsed with distilled water prior to each experiment. The counter electrode was a 10 mm × 10 mm Ti sheet coated with IrO_2/Ta_2O_5. A saturated mercury sulfate electrode (MSE), filled with 0.6 M K_2SO_4, served as the reference electrode. All three-electrode potentials in this study are reported relative to the MSE for consistency. The electrolyte, with a total volume of 100 mL, consisted of deionized water, 0.08 M $CuSO_4$, and 1.53 M H_2SO_4, and all tests were conducted at 296 K. Cyclic voltammetry (CV) was carried out within a potential window of -1.2 to 0.4 V at a scan rate of 10 mV s^{-1}. Linear sweep voltammetry (LSV) was performed in the range of -0.3 to -1.6 V at a scan rate of 5 mV

s^{-1}. Chronoamperometric experiments were conducted at a constant potential of -0.7 V to investigate the electrochemical behavior of the additives. Electrochemical impedance spectroscopy (EIS) measurements were executed over a frequency range of 10^5 to 10^{-2} Hz, applying a 5 mV alternating current (AC) signal superimposed on a DC potential of -0.7 V. The obtained EIS data were analyzed using the Randles equivalent circuit model to extract key parameters, including solution resistance (R_s) and charge transfer resistance (R_{ct}). The apparent wetting behavior of the copper powder samples was evaluated by static water contact-angle measurements using an optical contact-angle analyzer (DSA-X ROLL, Guangzhou Beituo Science and Technology Co., Ltd.). The samples were uniformly compacted on a 20 mm × 20 mm × 0.5 mm stage using the same preparation procedure to form relatively flat powder beds. A 3 μL droplet of deionized water was deposited onto each powder-bed surface using a microsyringe, and the apparent static water contact angle was recorded under ambient conditions after the droplet profile had stabilized. To evaluate the antimicrobial efficacy, a nutrient agar medium was prepared by dissolving 0.6 g of tryptone, 0.6 g of sodium chloride, 0.3 g of yeast extract, and 0.9 g of agar powder in deionized water. Copper powder was added at a concentration of 5×10^{-4} g mL^{-1}, and the final volume was adjusted to 60 mL. The mixture was homogenized by thorough agitation. Subsequently, the medium was sterilized at 121°C for 20 min using an autoclave. Post-sterilization, the medium was aseptically poured into sterile Petri dishes and allowed to solidify. The solidified agar was then evenly inoculated with *Escherichia coli* or *Staphylococcus aureus*. Bacterial suspensions were uniformly spread onto the surface of the agar plates using a sterile spreader. The inoculated plates were then incubated at 310 K for 12 h. Following incubation, colony growth was examined and recorded to assess antimicrobial activity of the copper-containing medium. To obtain comparative data on soluble copper release under the agar-based antibacterial assay conditions, UCP-30Br and P-industrial powder were each incorporated into agar medium at the same copper dosage used in the antibacterial tests. Following a 12 h incubation, the agar blocks containing the copper samples were cut into small pieces and dispersed in 100 mL of deionized water at 298 K. The suspension was stirred at 300 rpm for 5 minutes to extract soluble copper species, and then filtered to remove residual solid particles. The resulting filtrate was analyzed using inductively coupled plasma mass spectrometry (ICP-MS, ELAN DRC-e, PerkinElmer, USA).

Reference

[1] W. Yang, S. Han, Y. Yin, Z. Wang, W. Shi, J. An, H. Wang, W. Xia, Achieving optimal dendritic morphology in ultralight copper powder production through hydrochloric acid intervention, J. Mater. Eng. Perform. 34 (2025) 6389-6399, https://doi.org/10.1007/s11665-024-09643-9.

[2] S. Han, Y. Yin, Y. Mise, S. Wang, W. Xia, X. Zhou, J. An, H. Wang, W.J.M. Yang, M.T. Brown, Mechanism of Cathode Residue Formation in Electrolytic Copper Powder Production and Mitigation Using Periodic Reverse Current, Metall. Mater. Trans. B 56 (2025) 2081-2096, https://doi.org/10.1007/s11663-025-03480-4.

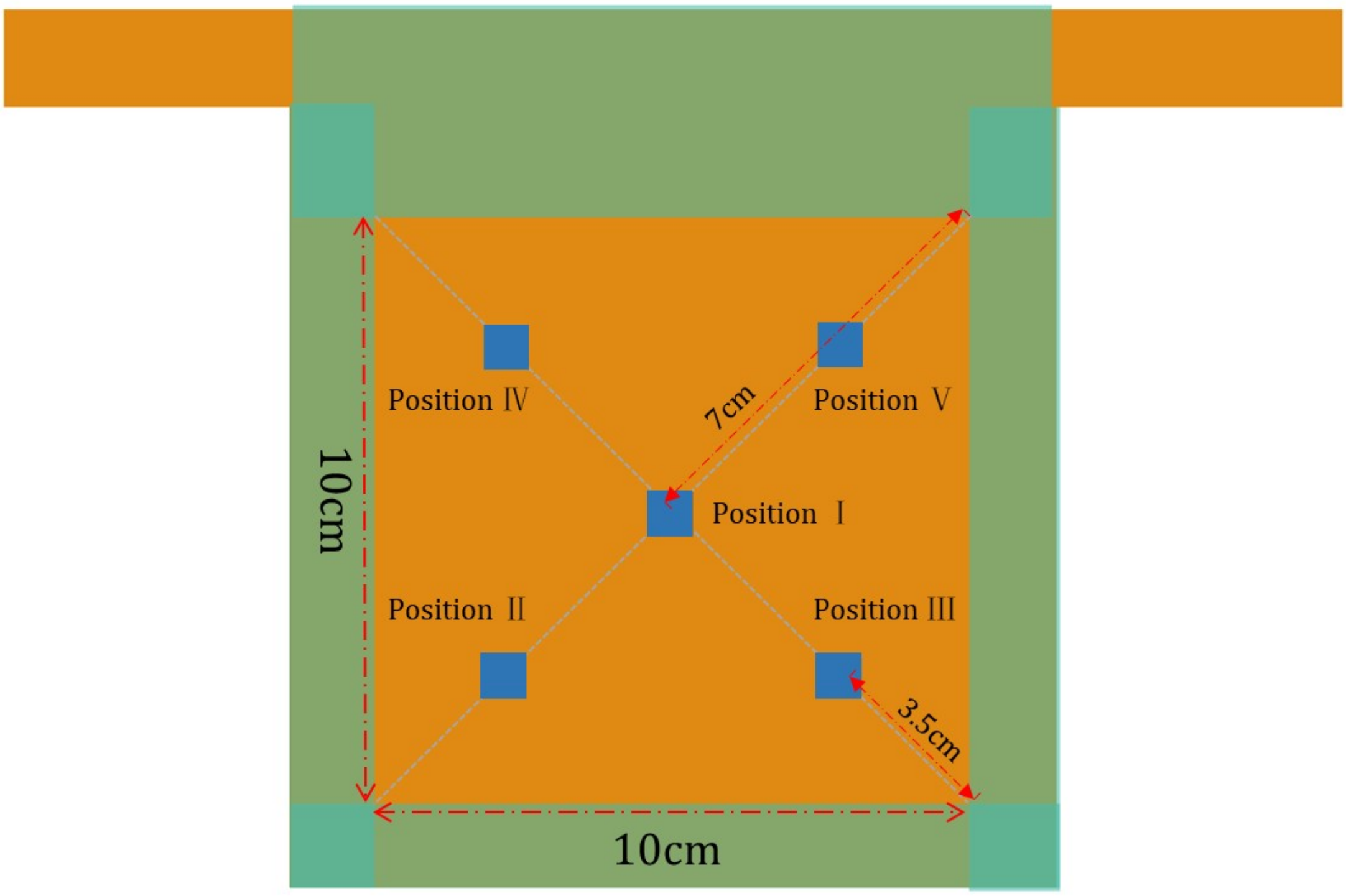

Fig. S2. Schematic illustration of surface roughness measurement locations on the electrode. The average roughness (Ra) values were calculated from five selected measurement points as indicated.

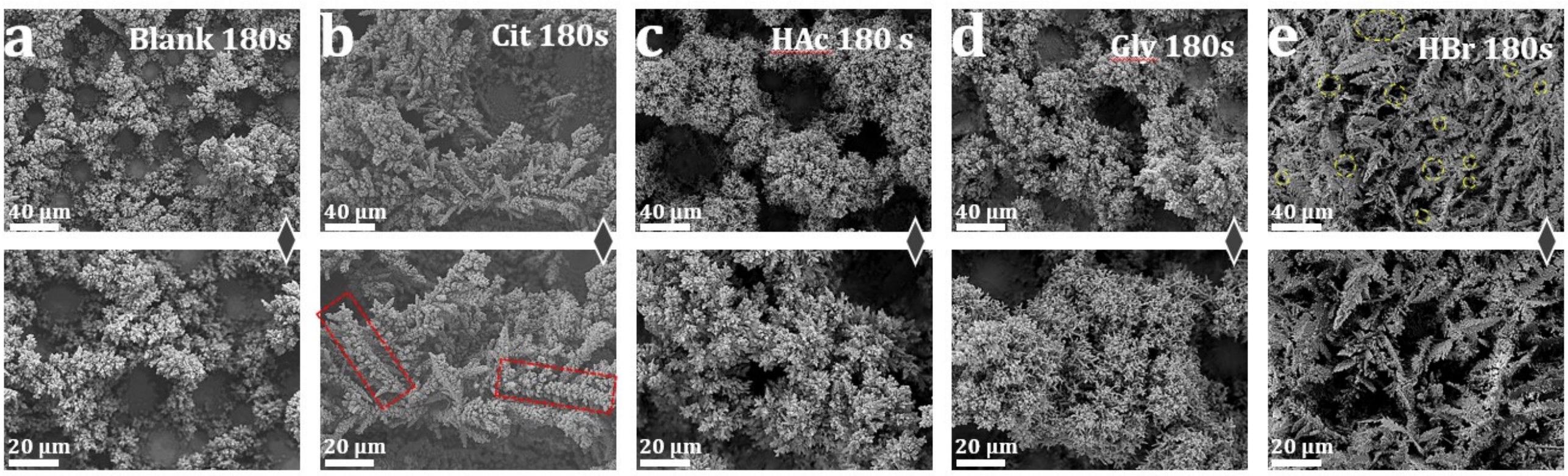

Fig. S3. Top-view SEM images of copper deposits obtained under condition A with different additives after 180 s of electrodeposition: (a) without additive, (b) additive Cit, (c) additive HAc, (d) additive Gly, (d) additive HBr.

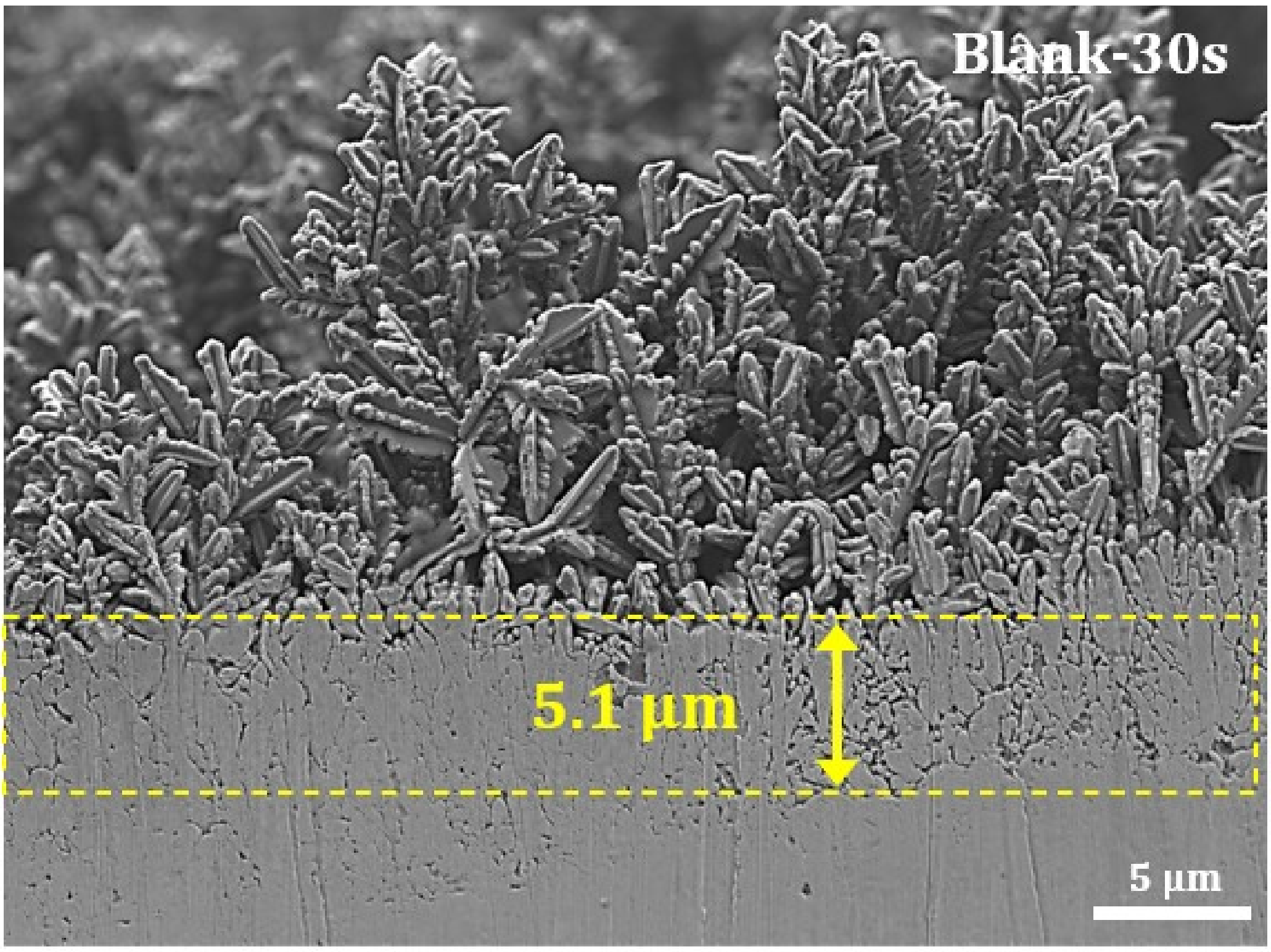

Fig. S4. Cross-sectional SEM image of copper deposit on Cu substrate obtained under condition A without additives after 30 s of electrodeposition.

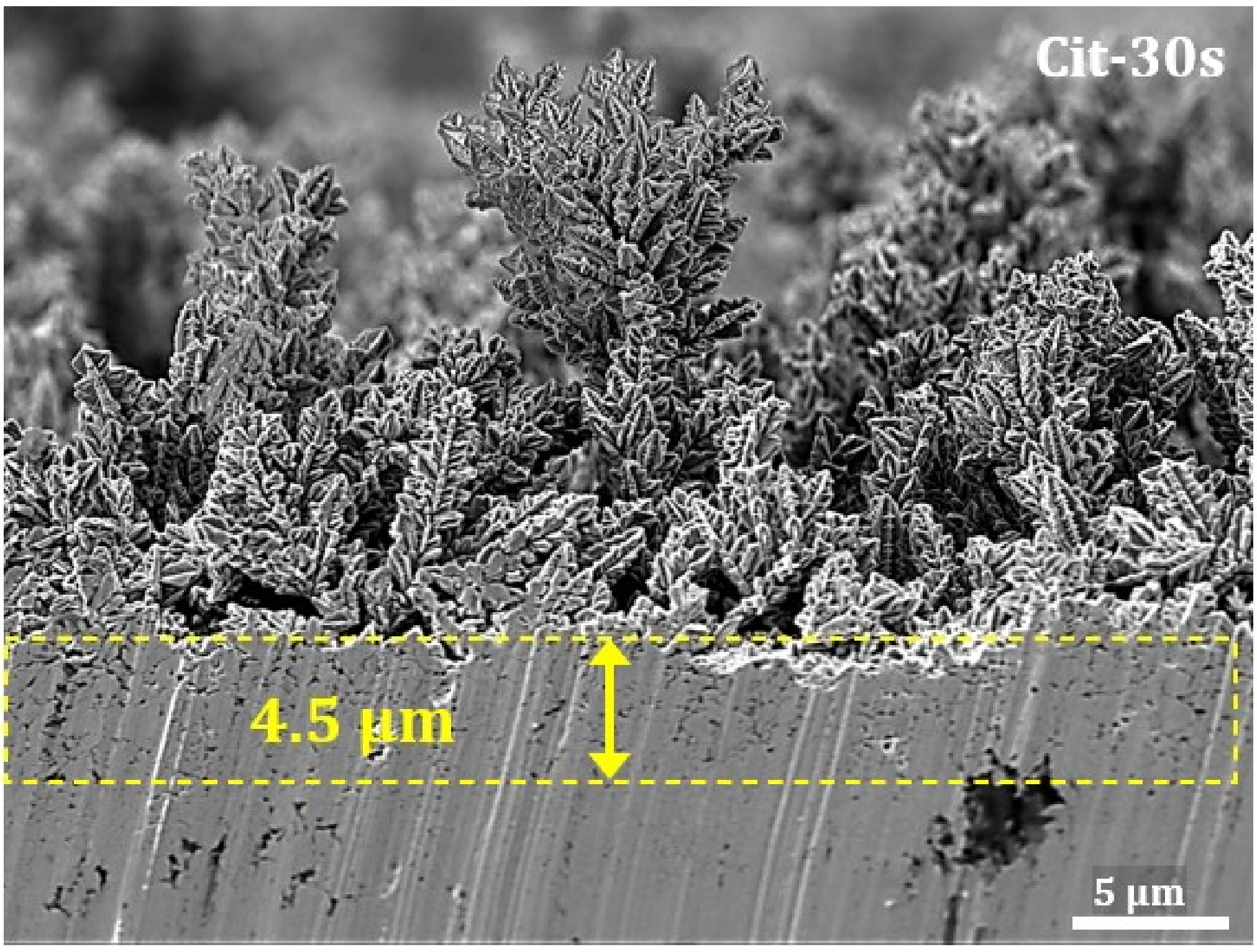

Fig. S5. Cross-sectional SEM image of copper deposit on Cu substrate obtained under condition A with Cit additive after 30 s of electrodeposition.

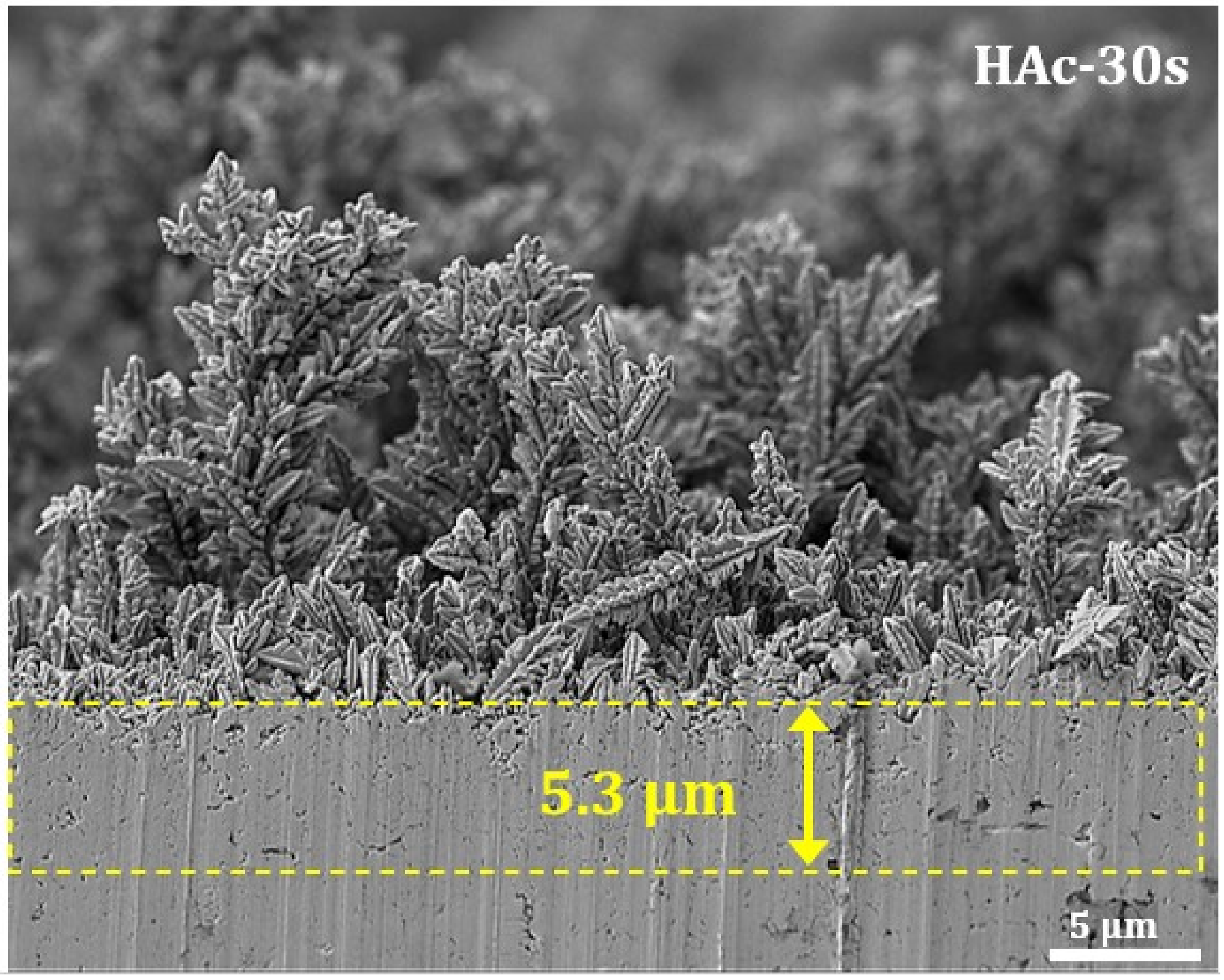

Fig. S6. Cross-sectional SEM image of copper deposit on Cu substrate obtained under condition A with HAC additive after 30 s of electrodeposition.

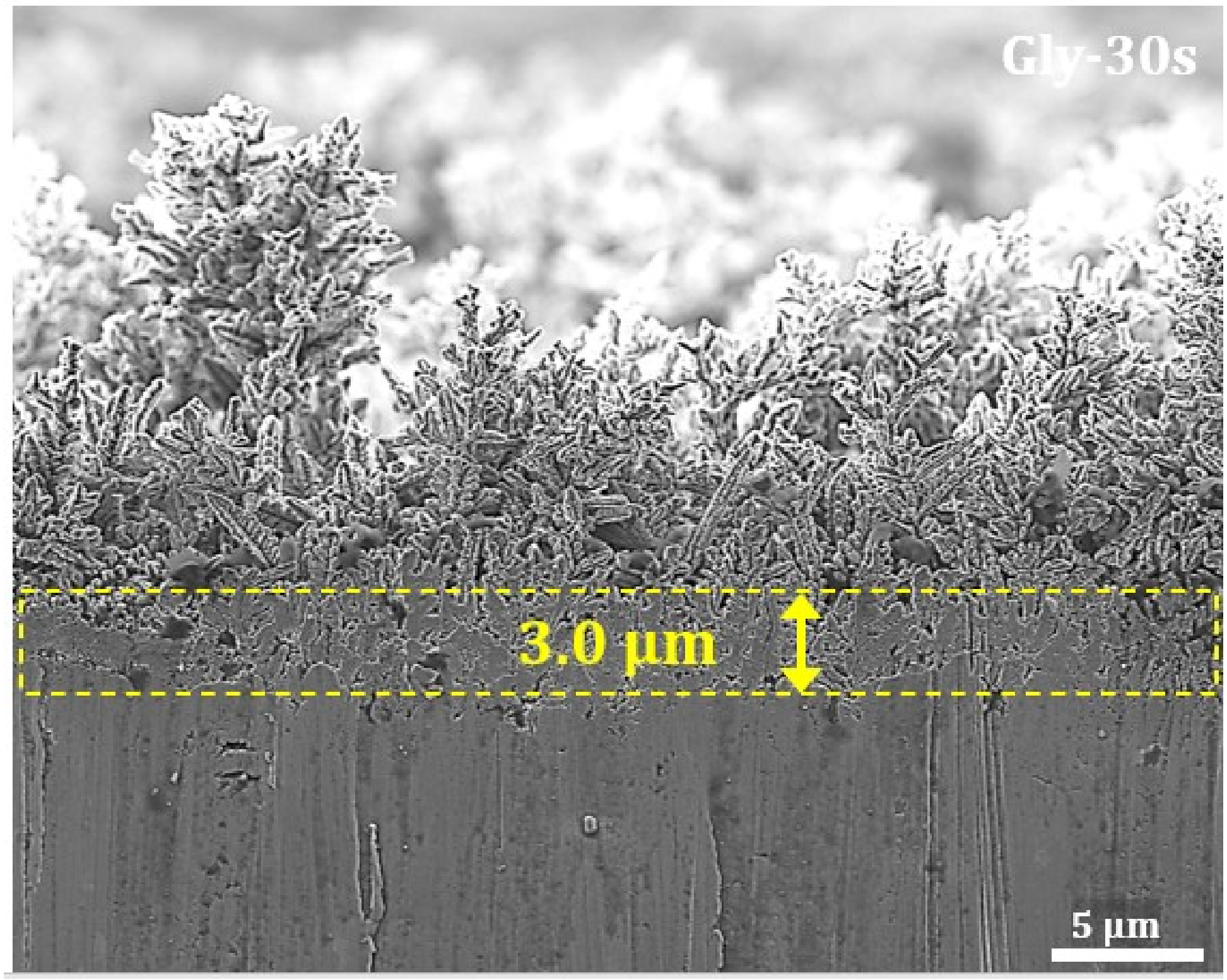

Fig. S7. Cross-sectional SEM image of copper deposit on Cu substrate obtained under condition A with Gly additive after 30 s of electrodeposition.

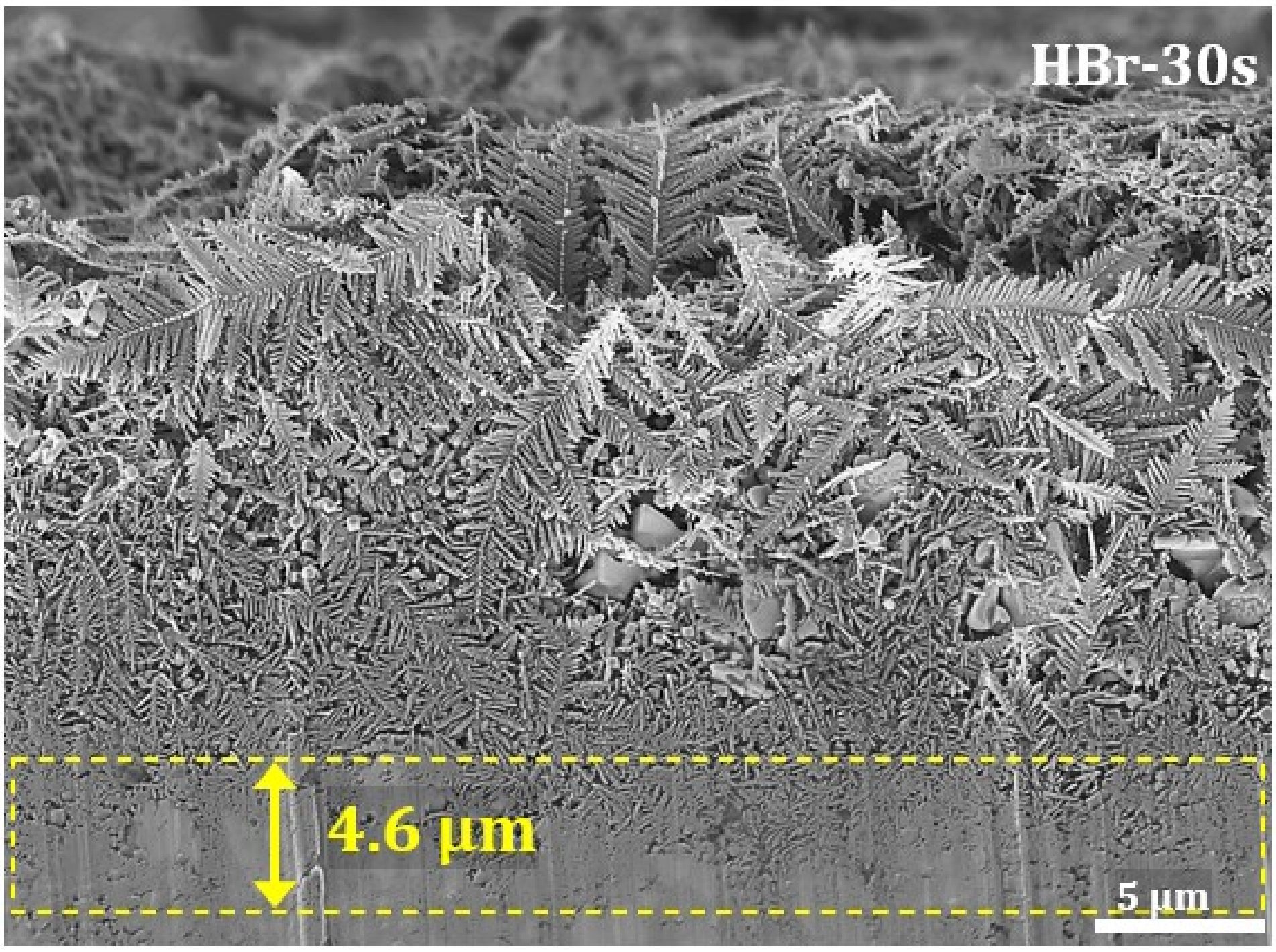

Fig. S8. Cross-sectional SEM image of copper deposit on Cu substrate obtained under condition A with HBr additive after 30 s of electrodeposition.

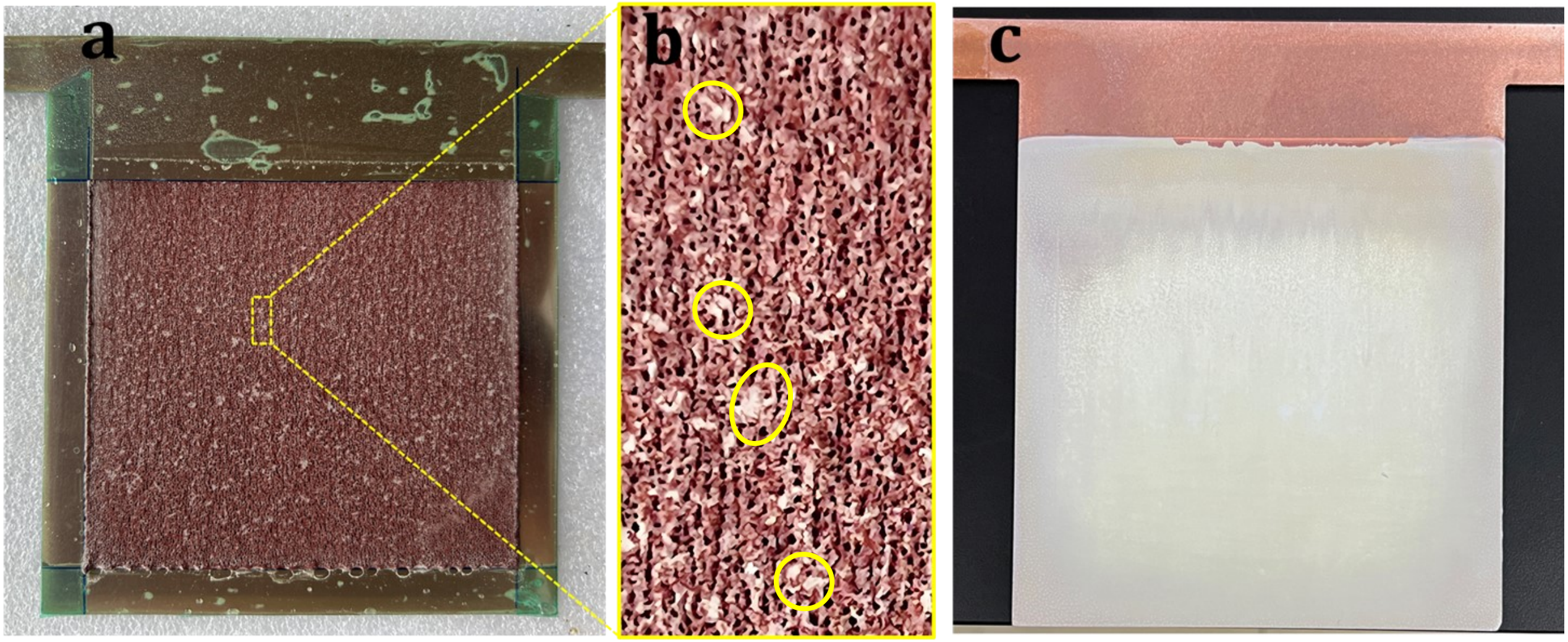

Fig. S9. Photographic images of electrodes after 3 min of electrodeposition under condition B: (a) overall view of the loaded cathode, (b) magnified view of the selected cathode region, and (c) overall view of the loaded anode.

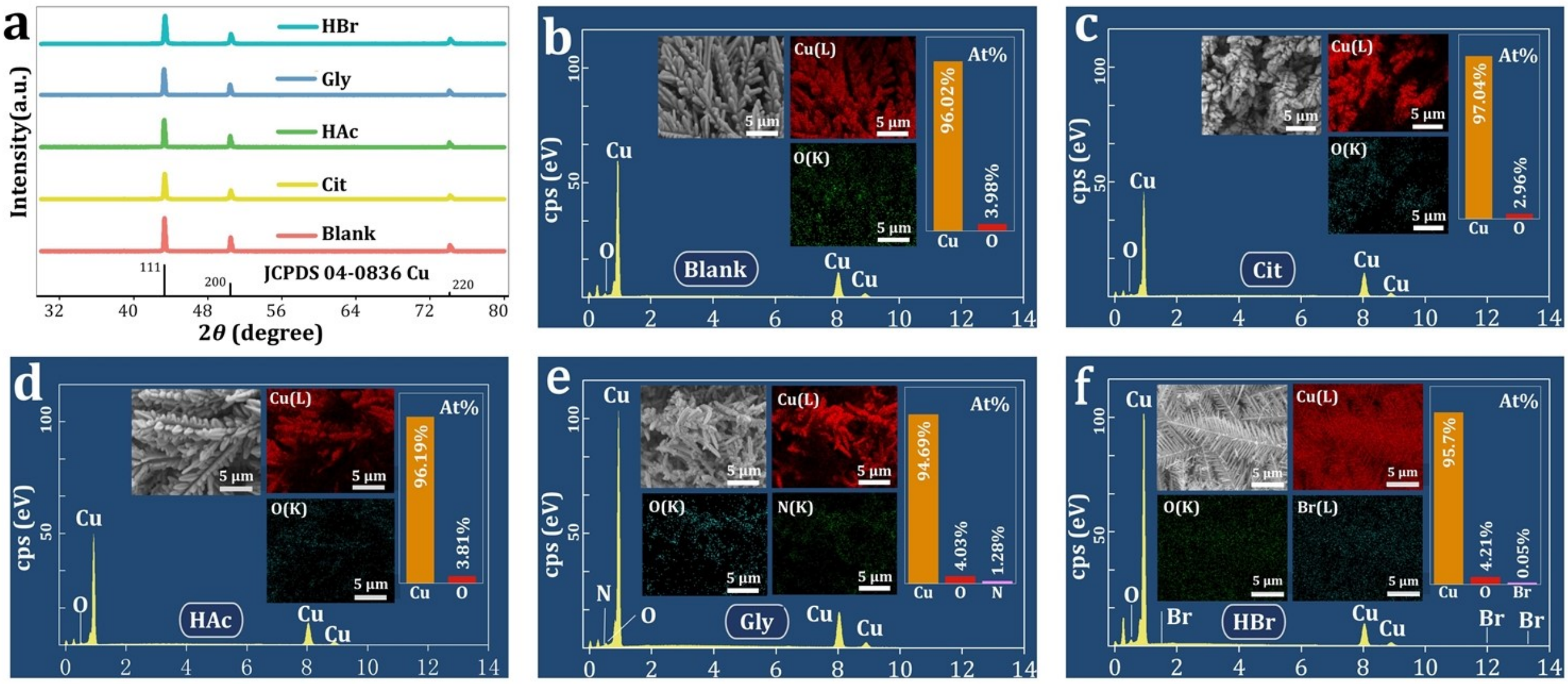

Fig. S10. (a) Powder XRD patterns and (b-f) EDS elemental analysis of powders prepared from electrolytes with various additives (as indicated) under condition A for 180 s.

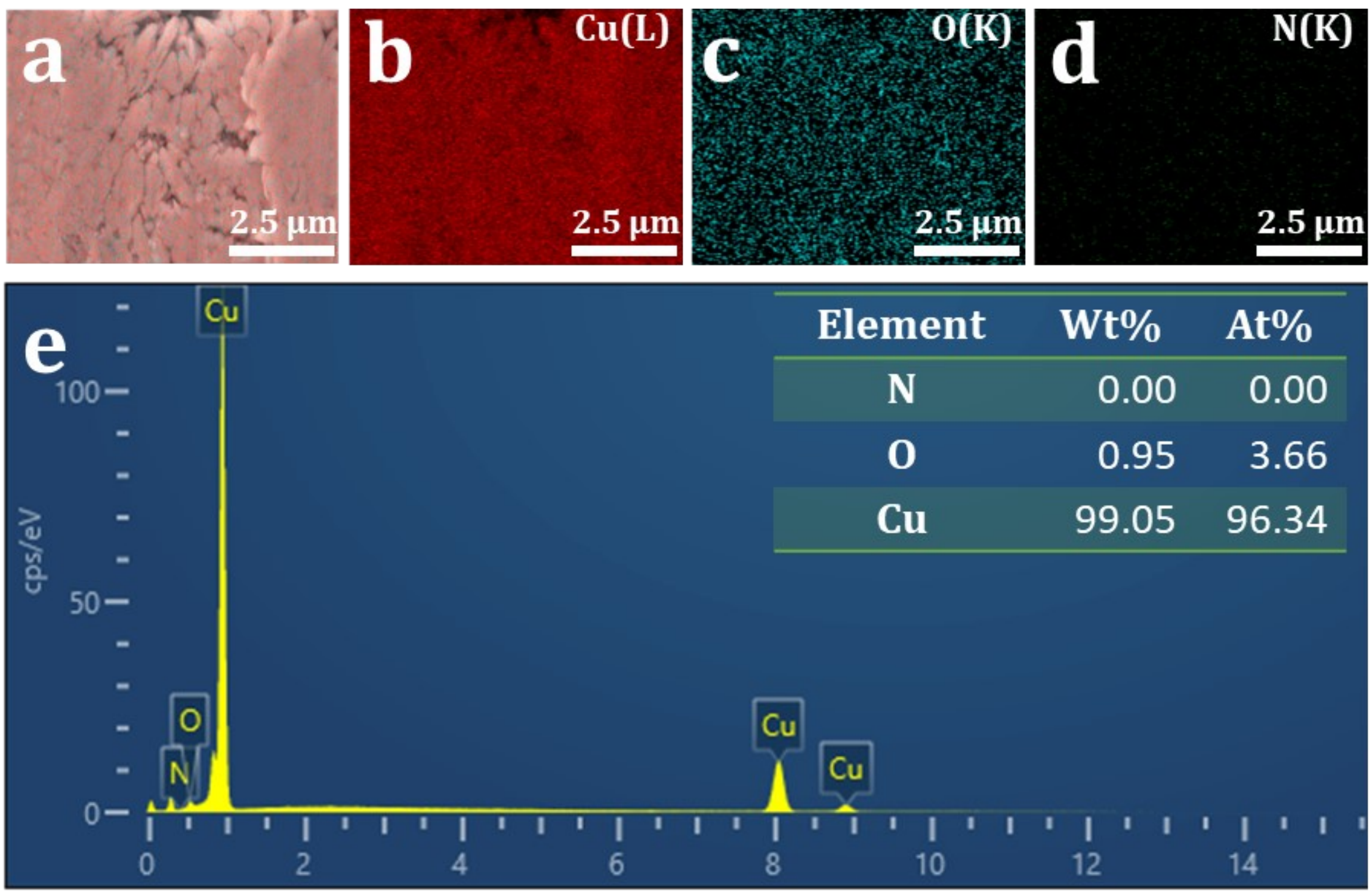

Fig. S11. (a) Cross-sectional SEM image, (b-d) corresponding elemental mapping, and (e) EDS spectrum of the Gly-assisted

copper deposit synthesized under condition A.

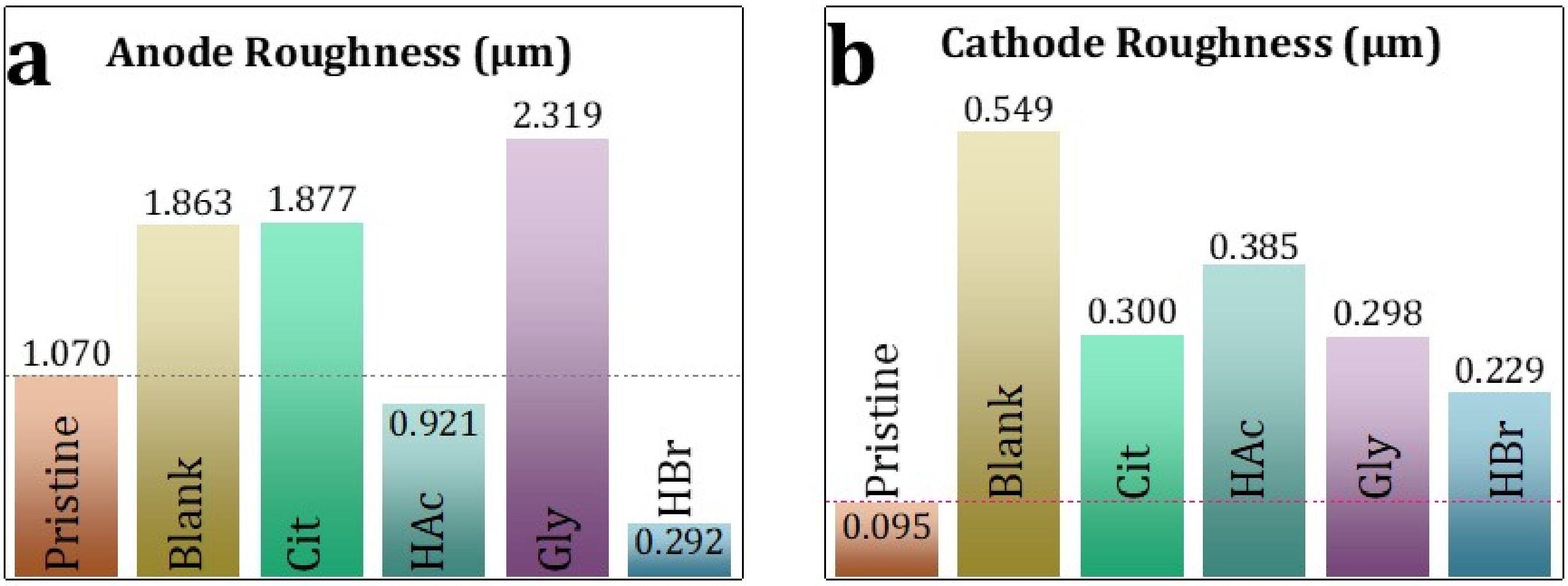

Fig. S12. Surface roughness analysis of post-scraping electrodes after 180 s of electrodeposition under condition A with different additives (as labeled): (a) cathode surface and (b) anode surface.

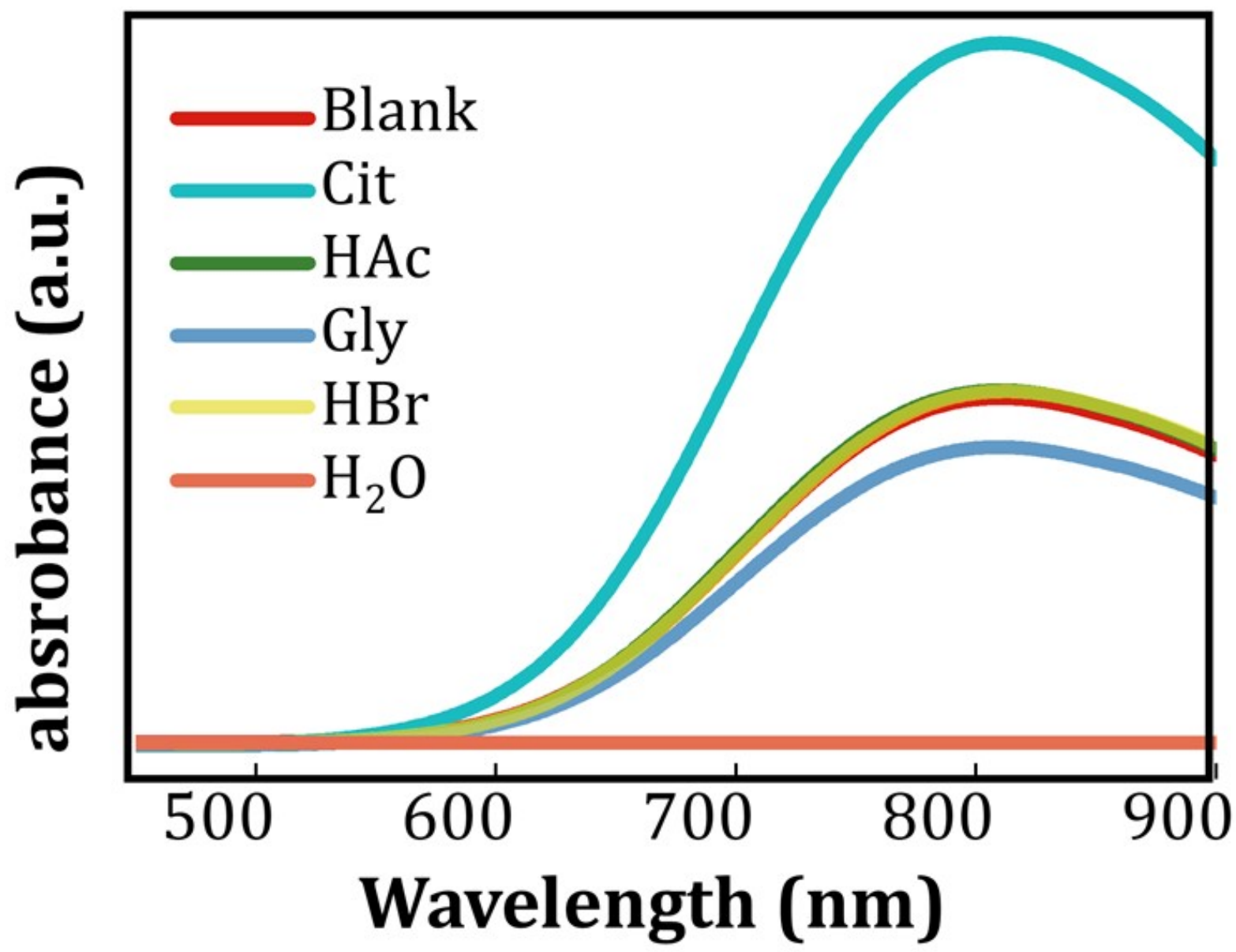

Fig. S13. UV-vis spectra of electrolyte systems containing 1.53 M H_2SO_4, 0.08 M $CuSO_4$, and 0.15 M specified additive (as

indicated) at 296 K.

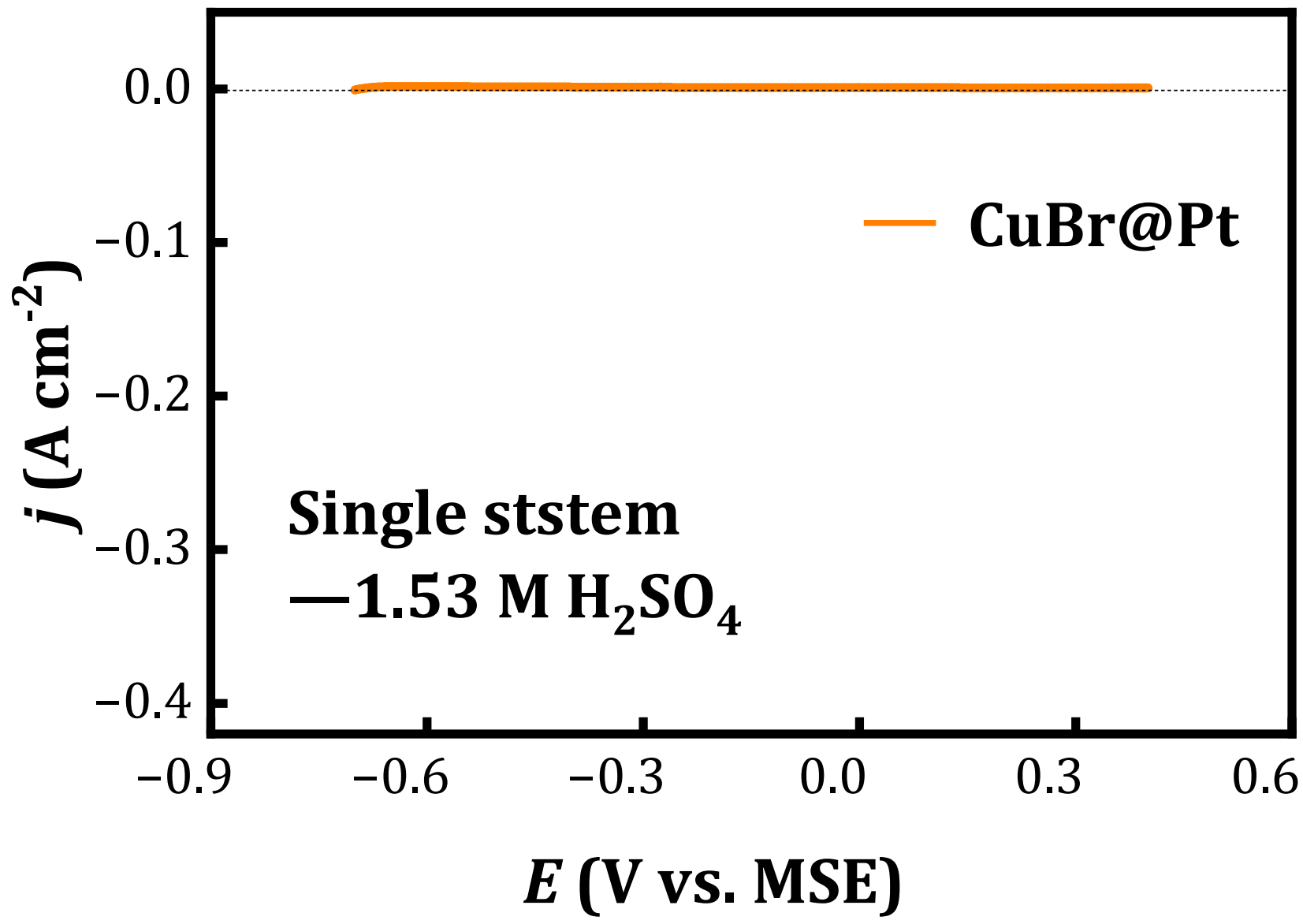

Fig. S14. CVs of the CuBr@Pt electrode in a single 1.53 M H_2SO_4 electrolyte system at 296 K, with a positive potential scan ranging from -0.7 to 0.4 V at a scan rate of 10 mV s^{-1}.

Preparation of CuBr-loaded working electrode: A homogeneous suspension was prepared by ultrasonically dispersing 0.01 g of CuBr (from Figure 7c), 40 μL of Nafion (5.0 wt.%), and 1 mL of ethanol/water solution (v/v = 1:1) for 30 min. The resulting suspension was then drop-cast onto a Pt sheet electrode (1.0 cm × 1.0 cm), maintaining a controlled CuBr mass loading of 10 mg cm^{-2}.

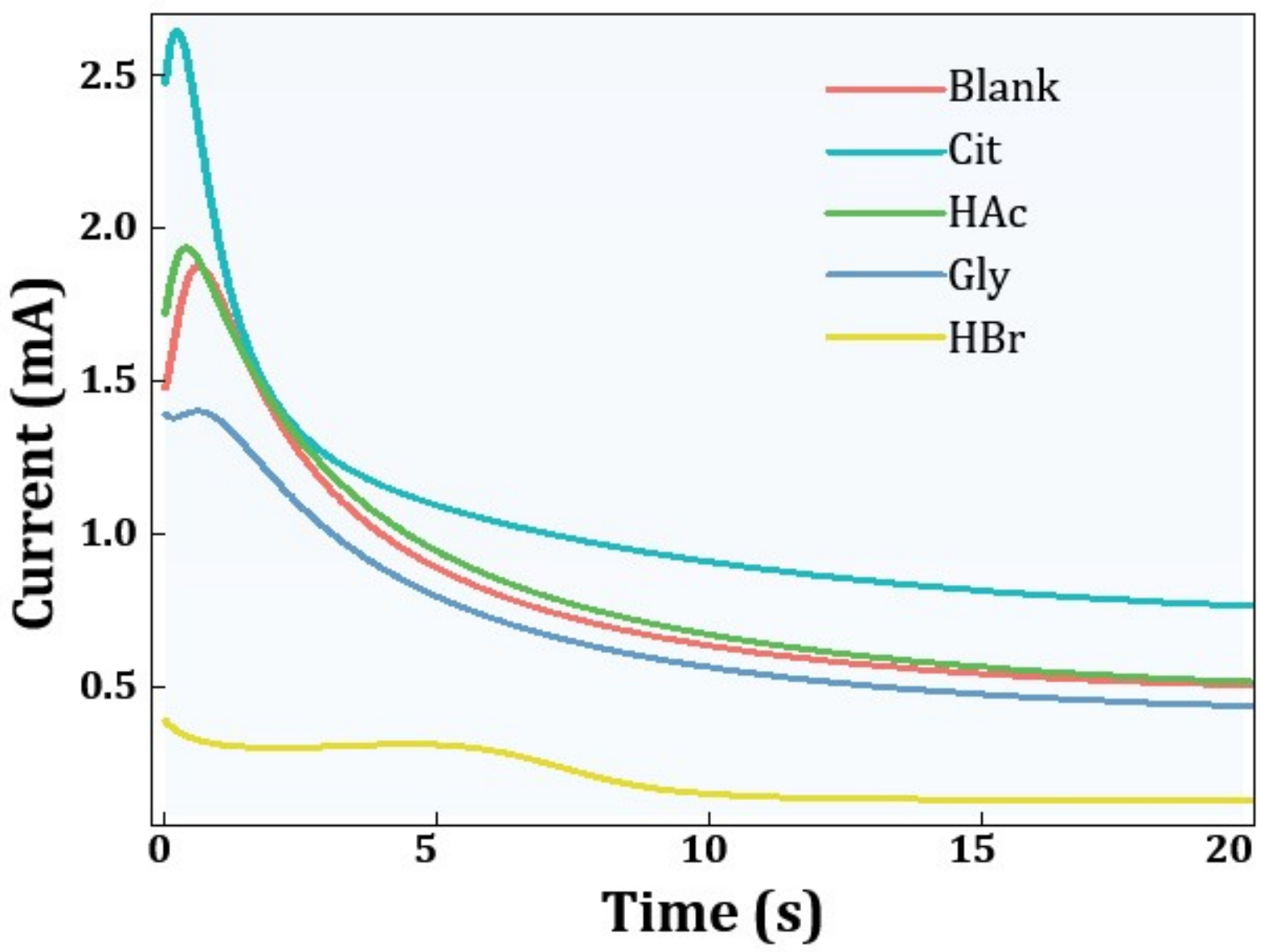

Fig. S15. Current-time transient curves for Cu electrodes in the same electrolytes at -0.7 V.

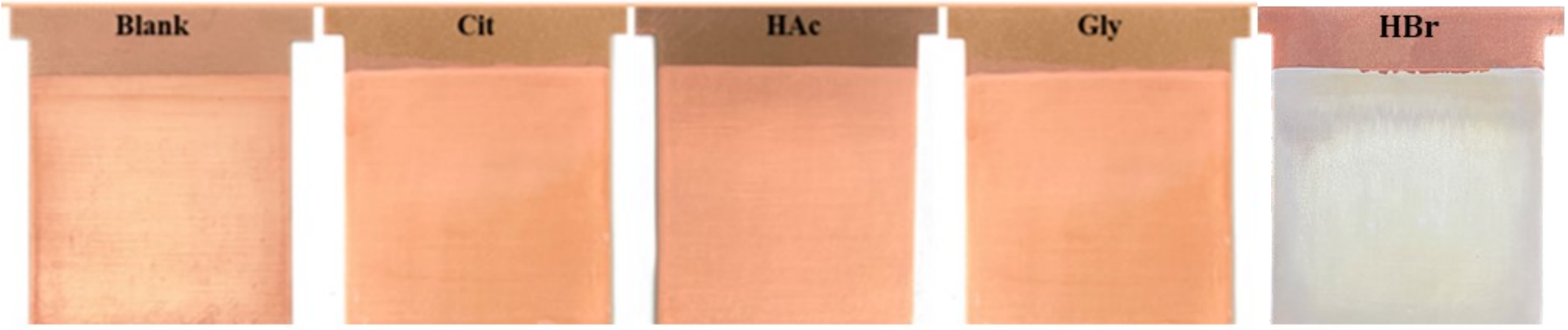

Fig. S16. Macroscopic images of anode surfaces after180 s of electrodeposition under condition A with different electrolyte additives (as indicated).

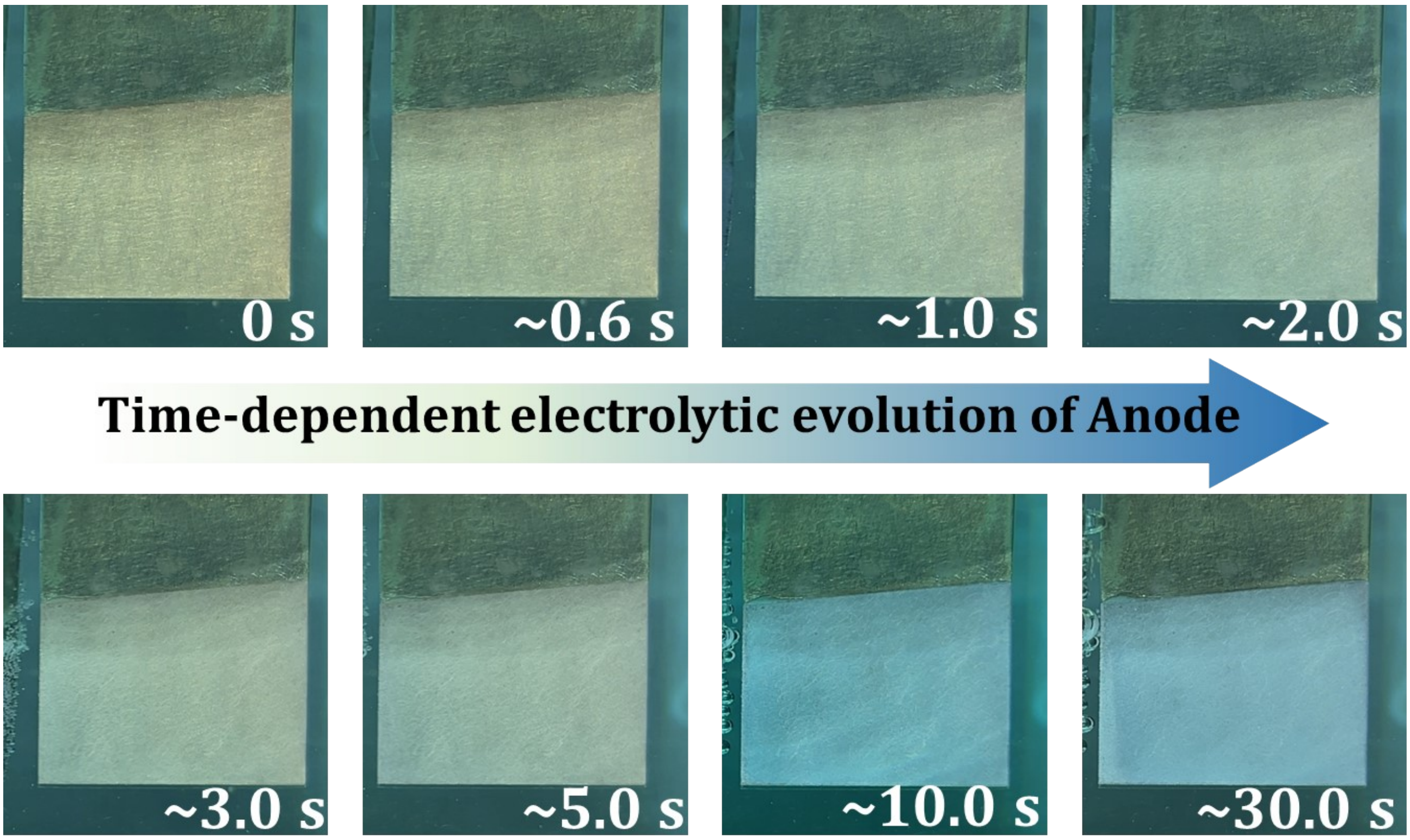

Fig. S17 Time-dependent macroscopic evolution of anode surfaces (1 cm × 1 cm) during electrolysis under condition B in a two-electrode configuration with copper as both anode and cathode.

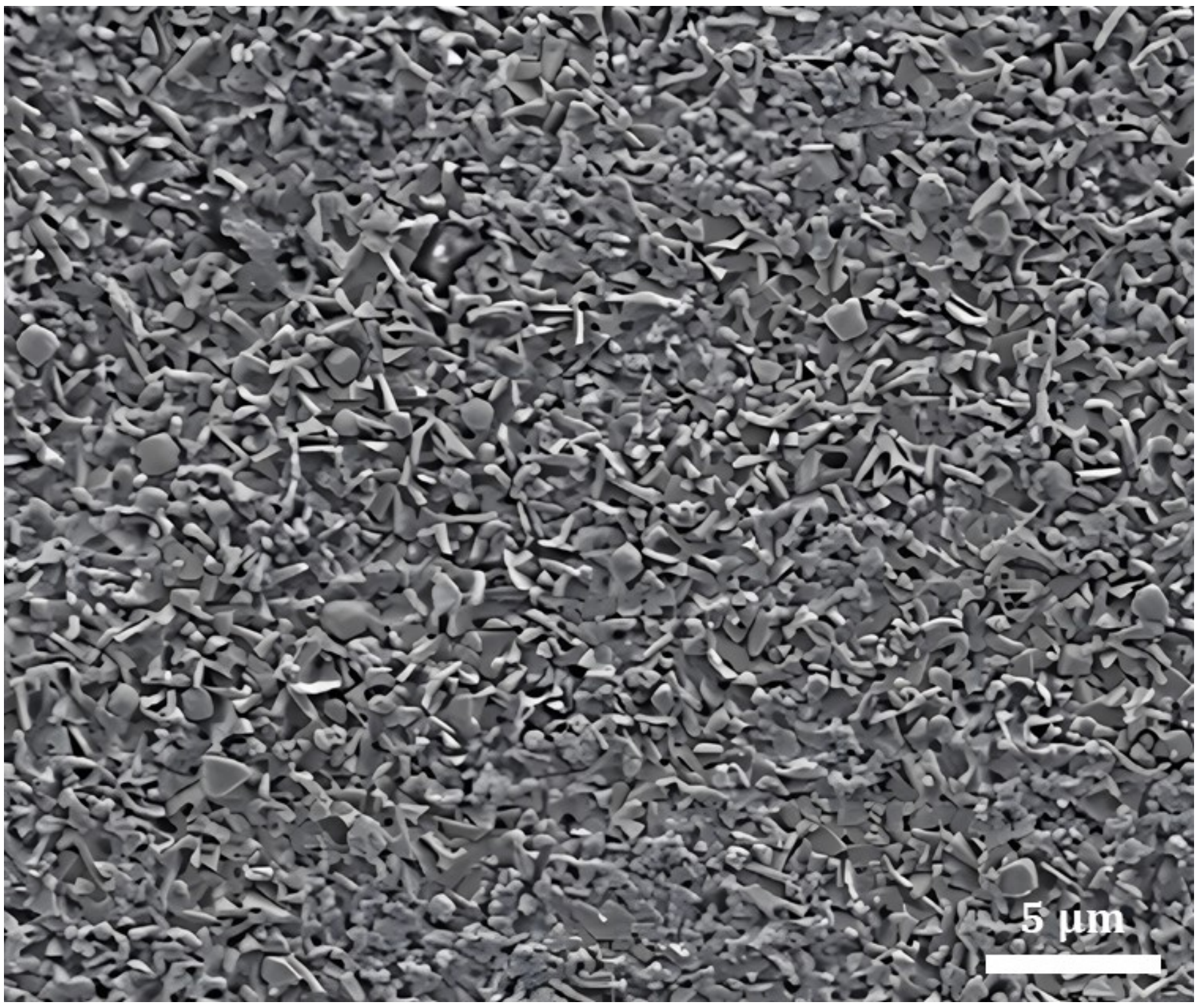

Fig. S18 SEM image of the anode surface after 180 s of electrodeposition under condition B.

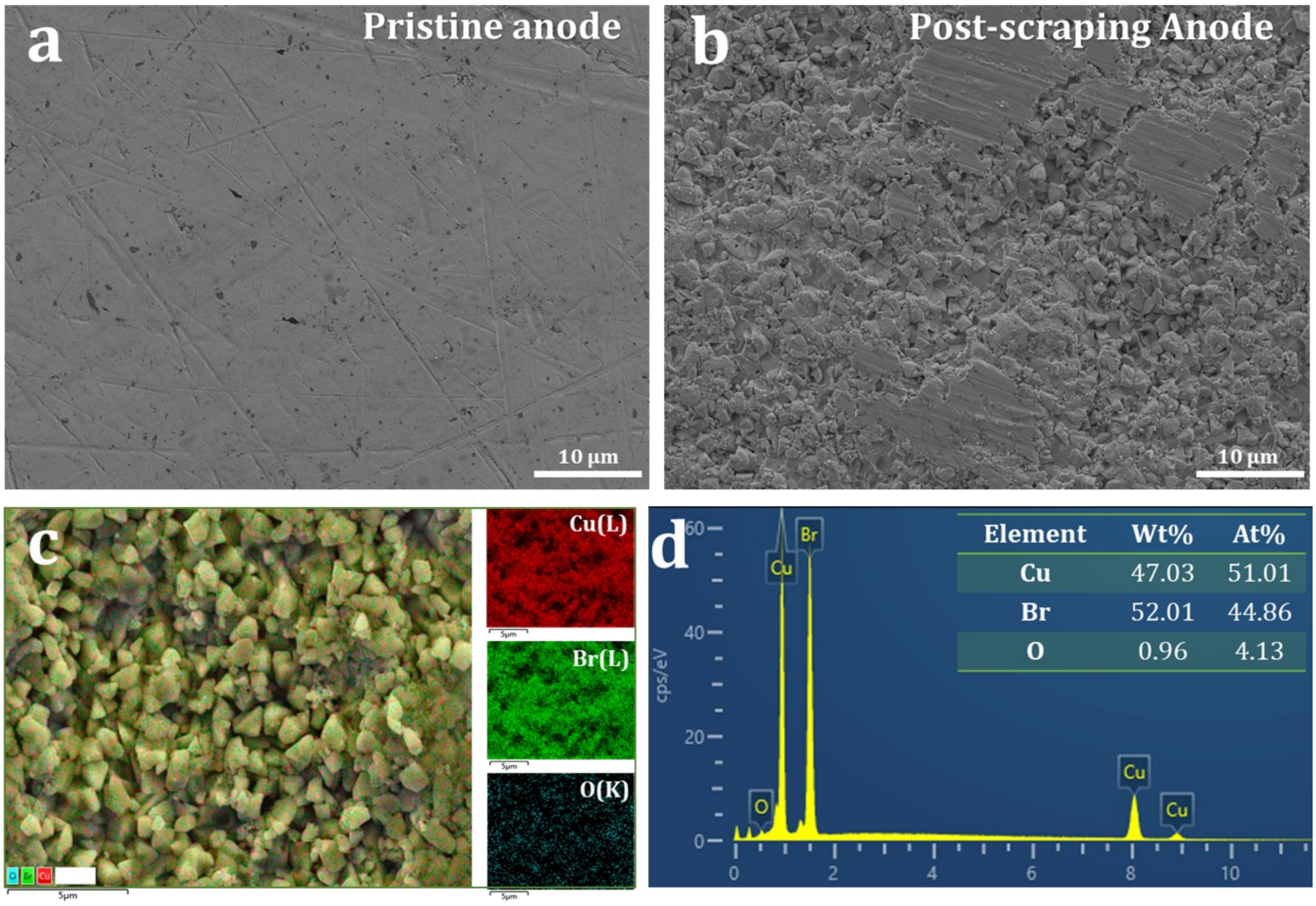

Fig. S19 (a) SEM image of pristine anode. (b) SEM image, (c) corresponding elemental mapping images, and (d) EDS spectrum of post-scraping anode surface after 180 s electrodeposition under condition B.

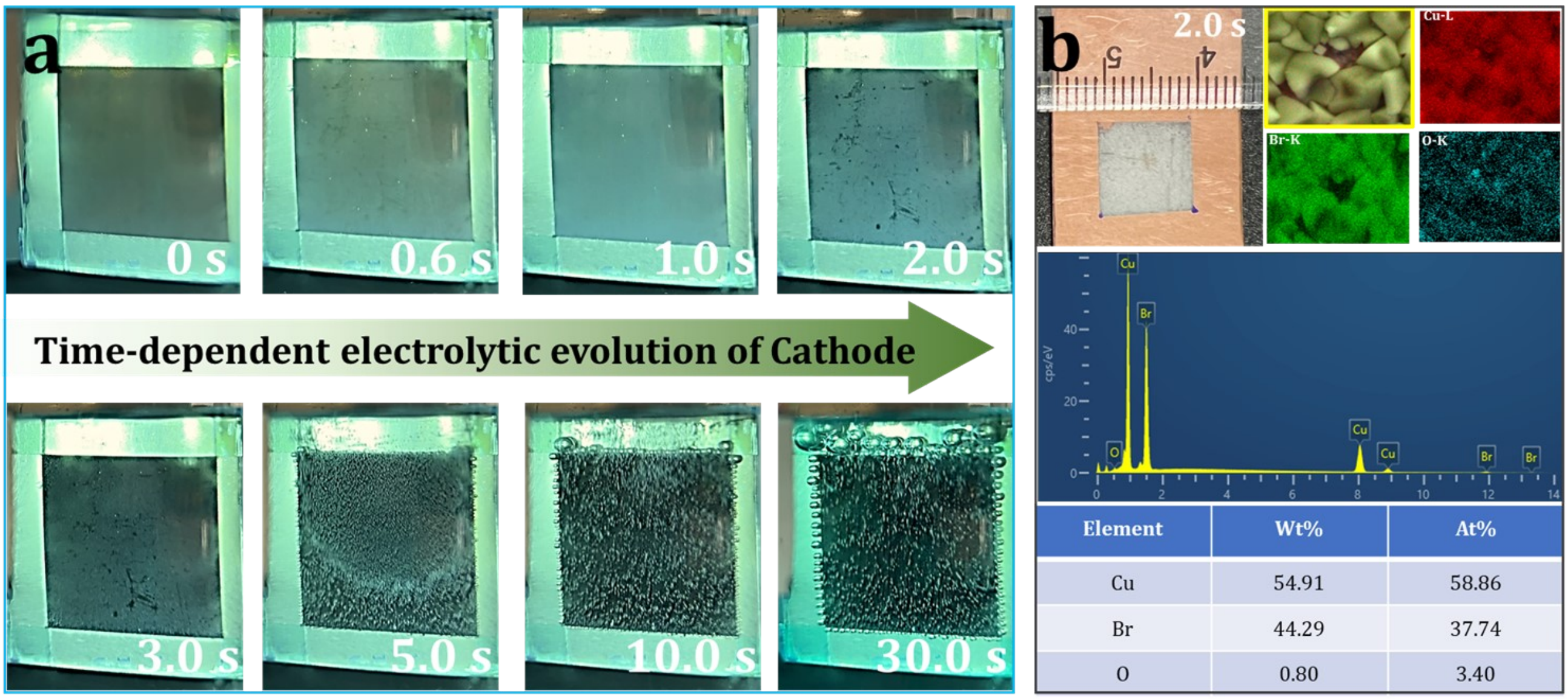

Element	Wt%	At%
Cu	54.91	58.86
Br	44.29	37.74
O	0.80	3.40

Fig. S20 (a) Time-dependent macroscopic evolution of cathode surfaces (1 cm × 1 cm) during electrolysis under condition B in a two-electrode configuration with copper as both anode and cathode. (b) Macroscopic image combined with SEM-EDS analysis and corresponding elemental mapping of cathode surface after deposition under condition B (2 s per deposition, 20 depositions total).

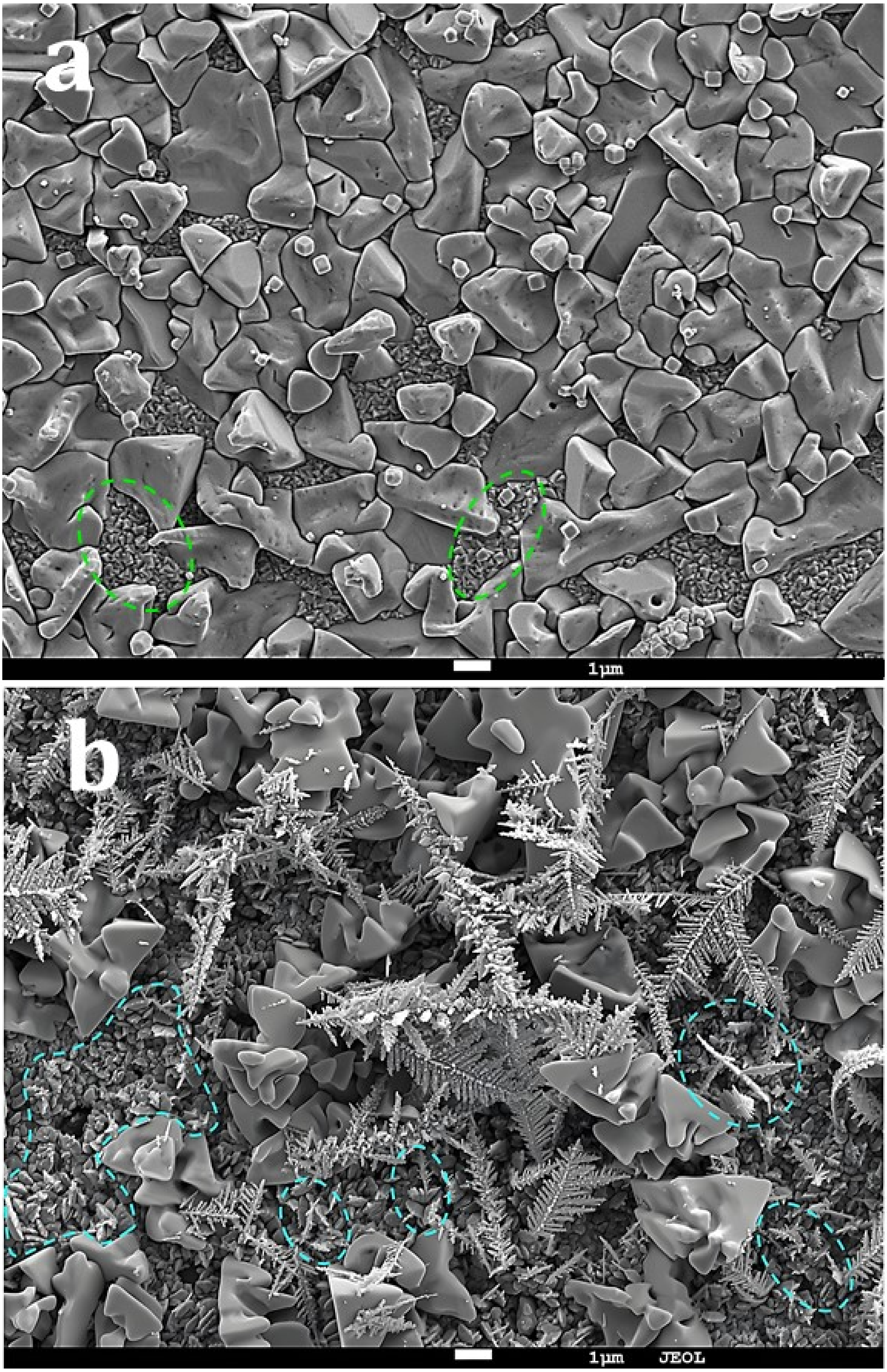

Fig. S21. SEM images of cathode surface after (a) 3 s and (b) 5 s of deposition under condition B.

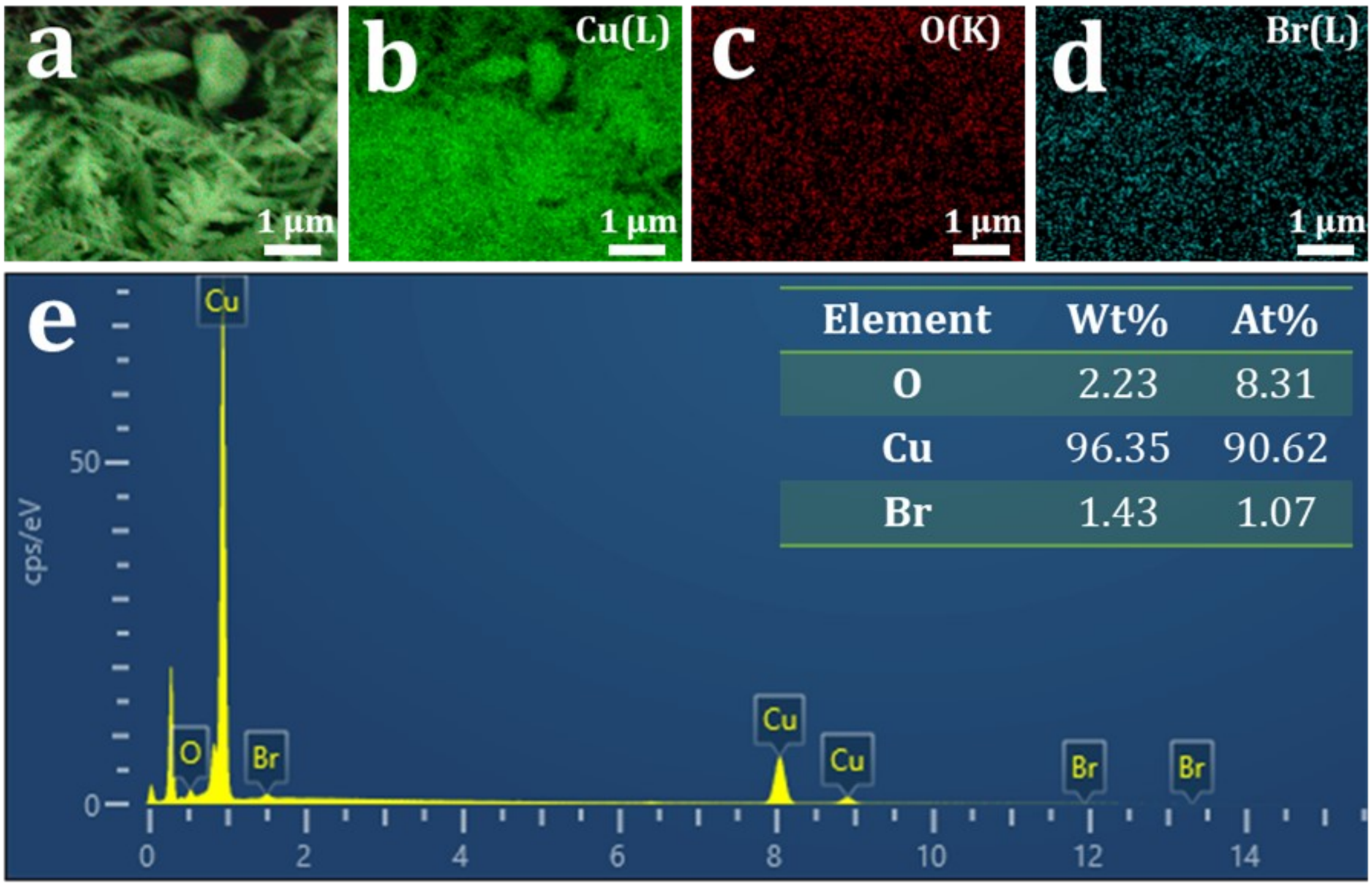

Fig. S22. Characterization of P-450Br: (a) SEM image, (b-d) corresponding elemental distribution maps, and (e) EDS spectrum.

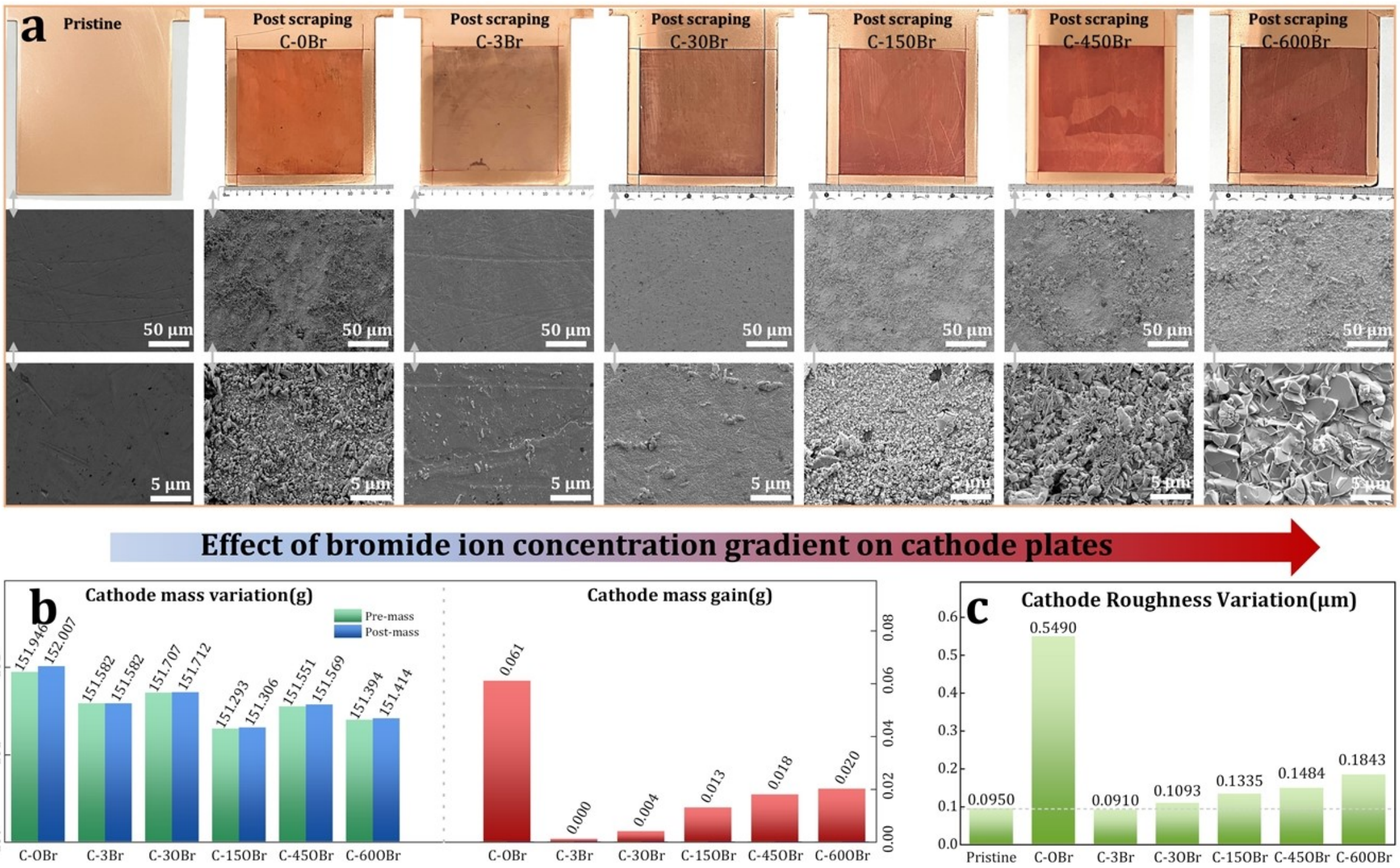

Fig. 23. (a) Macroscopic and microscopic images, (b) weight, weight gain, and (c) surface roughness of cathodes after deposition under condition C with various electrolyte additives (as indicated) and subsequent brushing treatment.

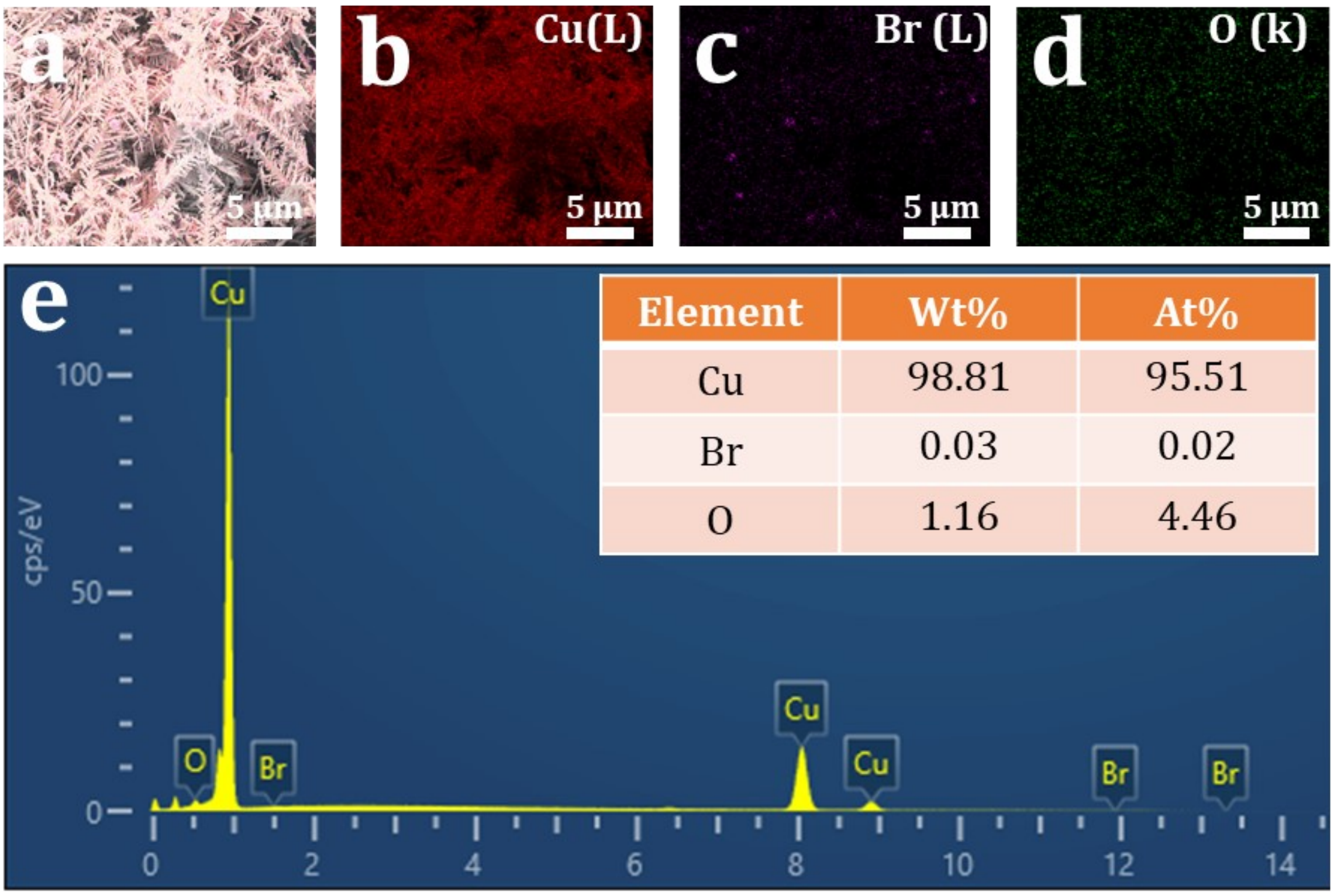

Fig. S24. (a) SEM image, (b-d) corresponding elemental distribution maps, and (e) EDS spectrum of UCP-30Br.

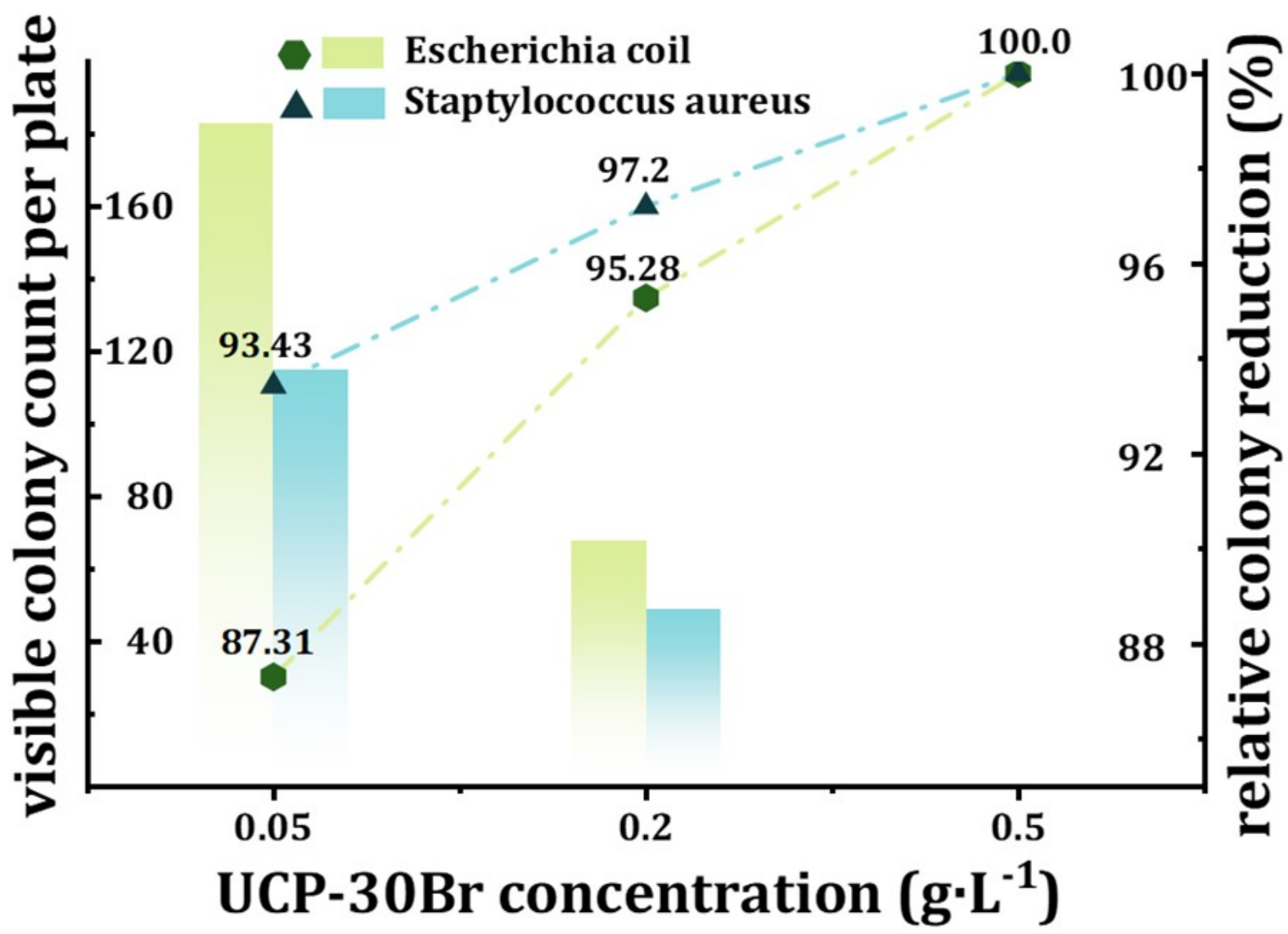

Fig.S25. Concentration-dependent reduction in visible bacterial colonies following UCP-30Br treatment. (a–d) Visible colony counts and relative reductions in colony number for *Escherichia coli* and *Staphylococcus aureus* after exposure to UCP-30Br at concentrations of 0.05, 0.20, and 0.50 g L^{-1} for 12 h.

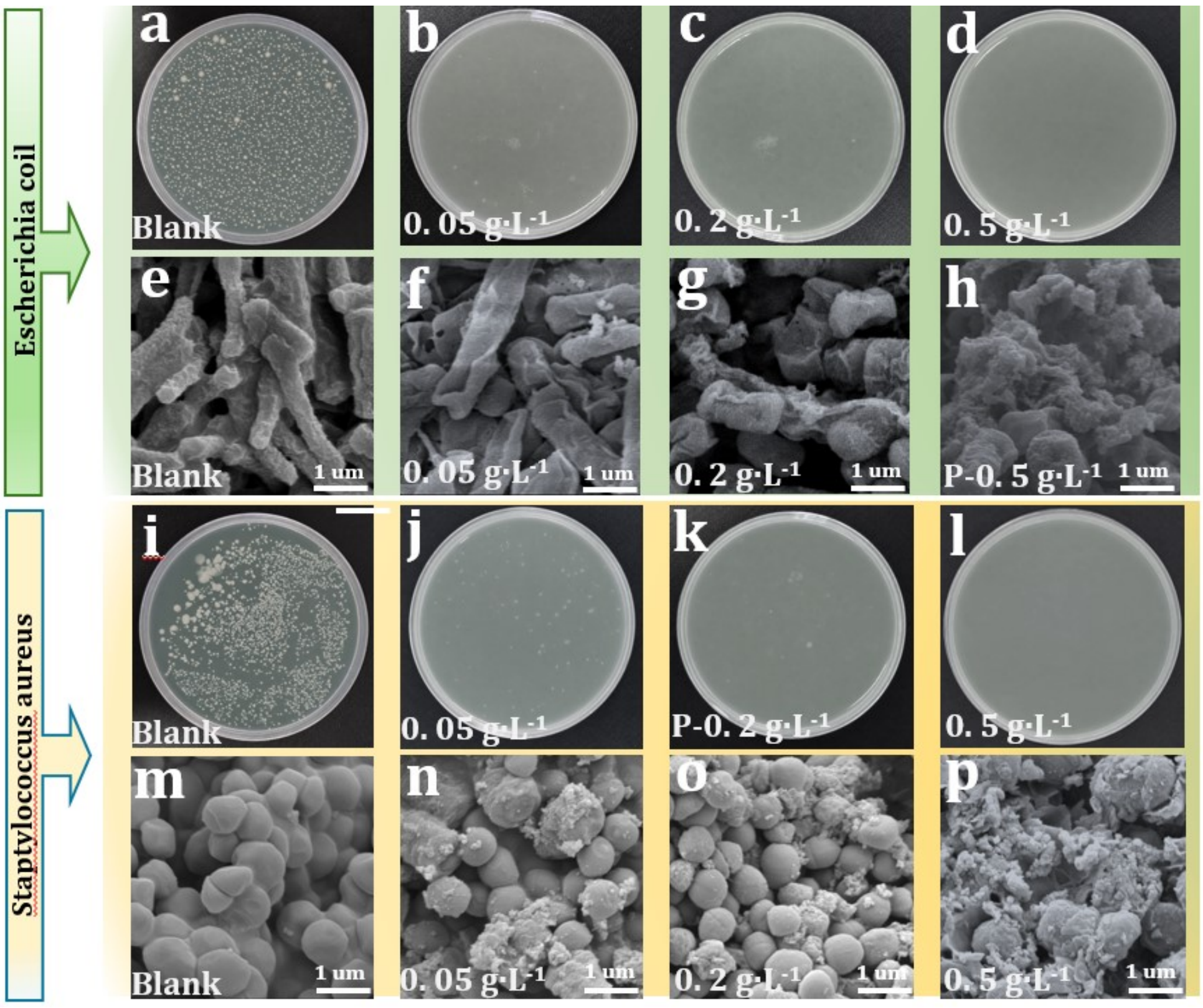

Fig. S26. Agar-plate and SEM images of *Escherichia coli* and *Staphylococcus aureus* after treatment with UCP-30Br for 12 h. (a–d) Agar-plate images and (e–h) corresponding SEM images of *Escherichia coli* treated with UCP-30Br at concentrations of 0, 0.05, 0.20, and 0.50 g L^{-1}, respectively. (i–l) Agar-plate images and (m–p) corresponding SEM images of *Staphylococcus aureus* treated under the same conditions.

Table S2 Fitted values of the EIS data (calculated from Fig. 6c).

Sample	R_s (Ω cm^{-2})	R_{ct} (Ω cm^{-2})
**Blank**	1.66	43.77
**Cit**	1.786	24.09
**HAc**	1.775	32.91
**Gly**	1.915	34.44
**HBr**	1.812	30.97

Table S3. ICP-MS quantification of soluble copper released from UCP-30Br and P-industrial in agar medium after 12 h incubation.

Sample	Soluble copper concentration (mg/L)
UCP-30Br	25.40
P-industrial	11.70
Deionized water (Blank)	0.0008
